# A new reduced-morphology model for CA1 pyramidal cells and its validation and comparison with other models using HippoUnit

**DOI:** 10.1038/s41598-021-87002-7

**Published:** 2021-04-07

**Authors:** Matus Tomko, Lubica Benuskova, Peter Jedlicka

**Affiliations:** 1 Centre for Cognitive Science, Department of Applied Informatics, Faculty of Mathematics, Physics and Informatics, Comenius University in Bratislava, 842 48 Bratislava, Slovakia; 2 ICAR3R—Interdisciplinary Centre for 3Rs in Animal Research, Faculty of Medicine, Justus-Liebig-University, Rudolf-Buchheim-Str. 6, 35392 Giessen, Germany; 3 Institute of Clinical Neuroanatomy, NeuroScience Center, Goethe-University Frankfurt, Frankfurt am Main, Germany

**Keywords:** Biophysical models, Computational models, Computational neuroscience, Computational science, Computer science

## Abstract

Modeling long-term neuronal dynamics may require running long-lasting simulations. Such simulations are computationally expensive, and therefore it is advantageous to use simplified models that sufficiently reproduce the real neuronal properties. Reducing the complexity of the neuronal dendritic tree is one option. Therefore, we have developed a new reduced-morphology model of the rat CA1 pyramidal cell which retains major dendritic branch classes. To validate our model with experimental data, we used HippoUnit, a recently established standardized test suite for CA1 pyramidal cell models. The HippoUnit allowed us to systematically evaluate the somatic and dendritic properties of the model and compare them to models publicly available in the ModelDB database. Our model reproduced (1) somatic spiking properties, (2) somatic depolarization block, (3) EPSP attenuation, (4) action potential backpropagation, and (5) synaptic integration at oblique dendrites of CA1 neurons. The overall performance of the model in these tests achieved higher biological accuracy compared to other tested models. We conclude that, due to its realistic biophysics and low morphological complexity, our model captures key physiological features of CA1 pyramidal neurons and shortens computational time, respectively. Thus, the validated reduced-morphology model can be used for computationally demanding simulations as a substitute for more complex models.

## Introduction

Biologically realistic compartmental modeling has become a standard and widely used method in neuroscience^1,2^. In combination with experiments, it represents a powerful tool for predicting and better understanding the complex electrophysiological behavior of neurons under physiological^3^ as well as pathological conditions^4^. However, despite considerable progress, a major challenge in the field has been that most published models have been tuned ad hoc for a limited set of experiments and therefore fail at generalizing the results to other experimental conditions^5^. We have recently developed a toolbox that supports the generalization of simulation results from one neuronal morphology to others^6^. Nevertheless, standardized tests for capturing a canonical set of experimental features are urgently needed for all nerve cell types. Accordingly, the HippoUnit test battery has recently been developed to address this problem for CA1 pyramidal cells^7^. So far it has been successfully applied to full-morphology models. However, simulations of long-lasting neuronal dynamics, occurring on the time scale of tens of minutes, hours, or days (e.g., long-term synaptic plasticity or homeostatic synaptic and intrinsic plasticity), are often not feasible in models with full morphology and detailed biophysics. Therefore, here we aimed to develop a new reduced-morphology model that would decrease computational times as compared to full-morphology models while preserving their biophysical realism.

CA1 pyramidal neurons are one of the most studied cell types in the brain. Many computational models of CA1 pyramidal cells have provided insights into their electrophysiological behavior and function. On ModelDB^8^, more than 130 model entries can be found (https://senselab.med.yale.edu/ModelDB/ModelList?id=258) including several biophysically and morphologically detailed models^9–16^. These models were created for simulations of selected experimental tests but some of them have been recently evaluated by using the standardized set of HippoUnit tests^7^. However, no reduced-morphology CA1 pyramidal neuron models have yet been exposed to such systematic testing.

Thus, in this work, we have used the battery of standardized HippoUnit tests to validate our CA1 pyramidal cell model with reduced morphology. For a comparison, we used a previously validated full-morphology model^17^ as a reference model and three published reduced-morphology models that have not been tuned before for any of the HippoUnit features as controls. The three reduced-morphology models have been tuned previously for the following specific purposes. The models by Cutsuridis et al. (2010) and Cutsuridis and Poirazi (2015) have been used in circuit models with realistic spike timing of several hippocampal neuron types with respect to theta rhythm (see details in Methods). These models simulated input pattern recall and storage in the CA1^18^, and long latencies of hippocampal cell activities in the entorhinal-hippocampal network due to theta modulated inhibition^19^, respectively. The model from Turi et al. (2015) has been used within a hippocampal circuit model that has predicted the role of disinhibition in goal-directed spatial learning^20^.

Here, we employ the HippoUnit tests to compare these published models to a new compartmental model with reduced morphology. We show that our model is able to account for the following six well-established characteristic anatomical and physiological properties of CA1 pyramidal cells (see Methods for details): (1) The reduced dendritic morphology contains all major dendritic branch classes. In addition to anatomy, the model reproduces also 5 key physiological features, including (2) somatic electrophysiological responses, (3) depolarization block, (4) EPSP attenuation (5) action potential (AP) backpropagation, and (6) synaptic integration at oblique dendrites. By measuring the runtimes of the models, we show that the reduced-morphology models shorten the computational times as compared to the full-morphology model. By comparing the runtimes among the reduced models, we confirm that differences in the numbers of compartments and in the ion channel content primarily dictate the length of the simulations.

## Methods

We used HippoUnit^7^, a Python test suite, for automatic and quantitative validation of compartmental models of neurons built in NEURON^21^. HippoUnit is based on NeuronUnit, an extensible SciUnit-driven library designed for the testing of neuron models^22^. HippoUnit implements standard experimental protocols that are run on hippocampal neuronal models, thus providing a systematic evaluation of model performance. We used the following tests that are available in HippoUnit: Somatic Features Test, Depolarization Block Test, Postsynaptic Potential Attenuation Test, Backpropagating Action Potential Test and Oblique Integration Test^7^.

In the HippoUnit, a set of target features is specified for each test expressed as the mean and standard deviation. The output of a model is compared to the experimental data via feature-based error functions. Error is expressed as the absolute difference between the model output and a mean value of a feature, divided by the standard deviation (Z-score)^23^. The overall model score for a given test is the average of the errors of all tested features^7^. All simulations in our study were performed under MS Windows 10, Python version 3.7 and NEURON version 7.7.2. Device specification was: 2.60 GHz CPU and 16 GB RAM.

### Computational models of CA1 pyramidal cell

**Figure 1:**
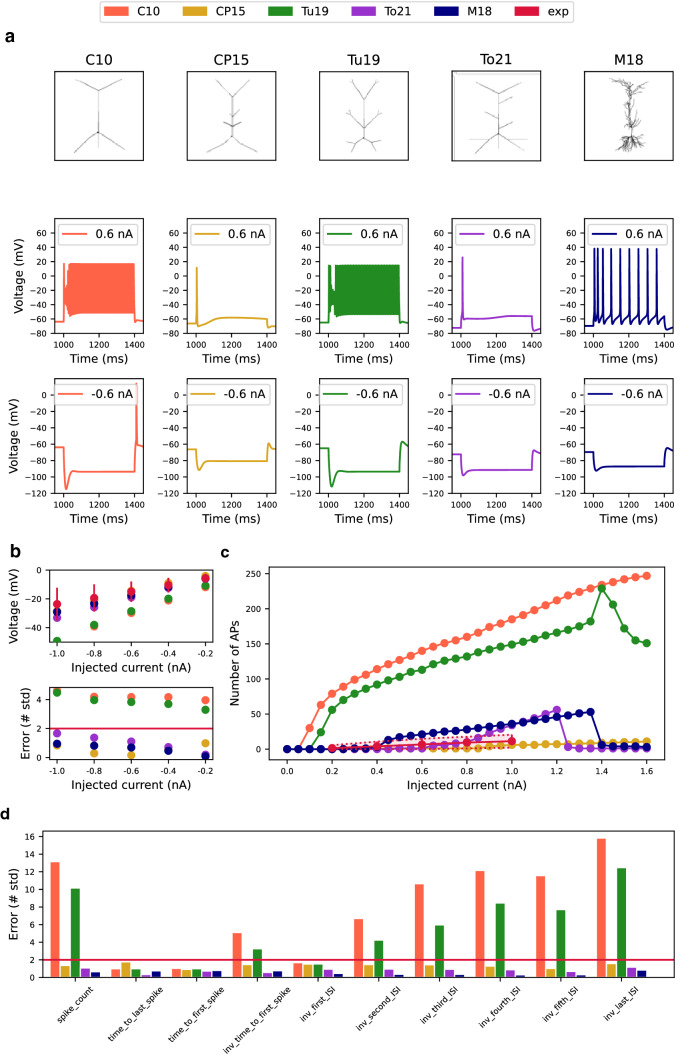
Validation of somatic voltage features, spike time features, and spike shape features using the Somatic Features Test (**a**) The first row shows the morphology of tested models. Reduced-morphology models are based on the simplified anatomy of the C10 model. For comparison, one well-tuned full-morphology M18 model was included. The second and third rows show the typical somatic response of the tested models to the positive and negative somatic current injections. (**b**) Voltage deflection of models across the different somatic current injections (top) and corresponding errors (bottom). The red horizontal line represents $sd=2$ and determines the maximum error accepted. (**c**) Number of fired APs as a function of injected current. The red line marked as experiment represents experimental values obtained from the Migliore et al. paper^17^. The red shaded area corresponds to twice the experimental standard deviation. (**d**) Model errors in selected spike event features expressed by the number of standard deviations. The red horizontal line represents $sd=2$ and determines the maximum error accepted.

Five compartmental models of the CA1 pyramidal cell were used for testing. Four models^17–20^ are available in the ModelDB repository (https://senselab.med.yale.edu/ModelDB/). Our new model has been optimized based on HippoUnit tests, the others were not and hence served as controls. The model of Migliore et al.^17^ has been previously tuned for selected features of the somatic feature test and was therefore included as a reference (benchmark) model. A further description of the models is given below. Their schematic architectures are shown in Fig. 1a.

#### Model of Cutsuridis et al.^18^—C10 model

This is a reduced-morphology model of the CA1 pyramidal cell that was used to model pattern encoding and retrieval in a small CA1 microcircuit. The model is available from ModelDB under accession number 123815. The model is composed of 15 sections, i.e., one for the soma, one for the axon, four for the basal dendrites, three for the apical dendritic trunk in the stratum radiatum and six for the dendritic tuft in the stratum lacunosum-moleculare (Fig. 1a). The model contains the following ion channels: Hodgkin-Huxley-like channel including both a sodium and a delayed rectifier potassium channel, a non-specific I_h_ channel, two additional types of potassium channels (K_A_, K_M_), three types of calcium channels (Ca_L_, Ca_R_, Ca_T_), two types of calcium activated potassium channels (K_Ca_, K_Cagk_), and a calcium pump / buffering mechanism. Biophysical properties were adapted from the Poirazi model^10,11^. For more details, see original paper^18^ and Table S3.

#### Model of Cutsuridis and Poirazi^19^—CP15 model

This is a similar reduced-morphology model of the CA1 pyramidal cell that was used in a computational study on how theta modulated inhibition can account for the long temporal windows in entorhinal-hippocampal loop. The model has the same morphology (Fig. 1a) as the C10 model described above, with one exception. Four sections connected to the apical trunk have been added to the model representing oblique dendrites. The model contains the same biophysical properties as the previous described model (see Table S3) that were adapted from the Poirazi model^10,11, 19^. Accession number in ModelDB is 181967.

#### Full-morphology model of Migliore et al.^17^—M18 model

To compare reduced-morphology models to a previously tuned realistic full-morphology model (Fig. 1a), we included the M18 model in our tests. This full-morphology model of the CA1 pyramidal cell is the result of a recent study that analyzed variability in the peak specific conductance of ion channels in individual CA1 neurons. A set of morphologically and biophysically accurate models was generated. The models are accessible from ModelDB under accession number 244688. The source code of the selected model is in a file named “cell_seed3_0-pyr-08.hoc”^17^. The model contains the following ion channels: a sodium channel, three types of potassium channels (K_DR_, K_A_, K_M_), a non-specific I_h_ channel, three types of calcium channels (Ca_L_, Ca_N_, Ca_T_), two types of calcium activated potassium channels (K_Ca_, K_Cagk_), and a calcium accumulation mechanism (see Table S3). The peak conductance of I_KA_ and I_h_ and the equilibrium potential of the passive current (e_pas) are calculated separately for each section according to its distance from the soma. The assignment of these values based on the distance from the soma is described by a linear function whose parameter values were fitted during the M18 model optimization process. The remaining conductances are distributed uniformly^17^.

#### Model of Turi et al^20^—Tu19

This reduced-morphology model of the CA1 pyramidal cell was used in a biophysical model of CA1 hippocampal region that simulates the place cells/fields dynamics. The active and passive properties are the same as in C10 model (see Table S3), however they were adjusted to achieve a more realistic attenuation of the AP backpropagation. The morphology (Fig. 1a) of the model is based on that of the C10 model. The model has two basal dendrites, each consisting of three sections. Oblique dendrites are modeled by two dendrites that are connected to the apical trunk in its proximal part. Each consists of four sections. Two dendrites of four sections together represent dendrites in the stratum lacunosum-moleculare^20^. Accession number in ModelDB is 246546.

#### Our new reduced-morphology model—To21

The reduced-morphology of our CA1 pyramidal cell model was inspired mainly by the C10 model. However, since the apical dendritic tree of this model consists only of the apical trunk (radTprox, radTmed, radTdist sections) and has no oblique branches, we extended the model by three thin oblique dendrites. With this improvement, we were able to better model synaptic integration on these branches (see Oblique Integration Test). In our model, the apical trunk of total length 400 µm consists of three interconnected sections that originate from the apical end of the somatic section. At the end of the apical trunk, two dendrites are attached. Each consists of three interconnected sections and together represents dendrites in the stratum lacunosum-moleculare (apical tuft). In addition, we added two branches to the basal dendritic tree. Furthermore, our model contains one section representing the soma and one section representing the axon. Basal dendrites are modeled by six sections, two for proximal stratum oriens (SO) dendrites and four for distal SO dendrites. The structure of the model is shown in Fig. 1a and in Table 1. Passive and active properties were adapted from the M18 model (Table S1–S3).

**Table 1:** Morphological structure of the To21 model.

Section	Section Lists	Num. of Segments	Diameter (µm)	Length (µm)
Soma	all, somatic	1	10	10
Axon	all, axonal	7	1	150
Proximal SO dendrite	all, basal	3	2	100
Distal SO dendrite	all, basal	7	1.5	200
Thick proximal SR dendrite	all, apical, trunk	3	4	100
Thick medium SR dendrite	all, apical, trunk	3	3	100
Thick distal SR dendrite	all, apical, trunk	7	2	200
Thin SR dendrites	all, apical, oblique	7	1	150
Thick SLM dendrite	all, apical	3	2	100
Medium SLM dendrite	all, apical	5	1.5	100
Thin SLM dendrite	all, apical	3	1	50

SO: stratum oriens, SR: stratum radiatum, SLM: stratum lacunosum-moleculare.

##### The model optimization

We used a trial-and-error approach to manually optimize our model. Instead of tuning the individual parameters randomly, we adapted the active and passive properties from the established full-morphology M18 model. With this strategy, we created a realistic hybrid model with reduced morphology. The goal of the optimization process was to achieve a final score of less than 2 in each HippoUnit test. During the optimization process, we manually adjusted the values of selected conductances. After each modification, we tested the model for the modified property. For the Depolarization Block Test, we adjusted the following conductances in the somatic section: g_Kap_, g_Kmb_, g_Kdr_, g_Nax_, g_Cal_, g_Cat_, g_KCa_. We used as initial values those listed in Table 1 in Bianchi et al.^12^. For the Backpropagating Action Potential Test, we adjusted the potassium and sodium conductances, specifically g_Kdr_, g_Kad_, g_Nax_, for the sections forming the apical trunk and the oblique dendrites. We modified the conductances on the basis of figure 7 in the paper of Golding et al.^24^ featuring bAP-tuned ion channel parameters.

#### Tests of modified reduced-morphology models (labeled as "vTo21" at the end of the model name; see Figs. 6, 7)

To test the hypothesis that changing the content of ion channels in reduced models can improve their physiological properties, we replaced the original set of channels in the C10, CP15 and Tu19 models with those from our To21 model (which were based on ion channels from the full-morphology M18 model). Subsequently, we tested the modified models using HippoUnit tests.

### Model validation by HippoUnit tests

The battery of HippoUnit tests has been described in detail before^7^. The Postsynaptic Potential Attenuation Test and the Backpropagating Action Potential Test (see below) required an explicitly defined list of sections forming an apical trunk. The apical trunk section list was defined as a continuous sequence of interconnected sections, the first section being connected to the soma and the last section being at the stratum radiatum / stratum lacunosum-moleculare boundary (about 350 µm from the soma). Similarly, the Oblique Integration Test required an explicitly defined oblique dendrites list. We created these lists for each tested model by hand. Here, we describe the details of tests used to validate the computational models in the current study.

#### Somatic features test

A total of 73 different somatic electrophysiological features are implemented in the Somatic Features Test. These include spike event features, spike shape features, and voltage features. In the first step of testing, the somatic membrane potential responses to somatic current injection of amplitudes from − 1.0 nA to 1.0 nA with a step size of 0.2 nA are obtained. Then, the Electrophys Feature Extraction Library (eFEL) of the Blue Brain Project^25^ is used for feature extraction from the voltage traces. The extracted somatic features are compared to experimental data obtained from sharp electrode recordings in adult rat CA1 pyramidal cells^17^. For each tested feature, the model error in the feature is expressed as multiple of the standard deviation. The mean feature error is calculated by averaging of errors over the different input step amplitudes. Finally, the resulting model score is computed by averaging of all errors^7^.

For closer comparison we selected 12 features based on which the M18 model was optimized^17^. They included voltage base, voltage deflection, spike count, time to last spike, time to first spike, 1.0 over time to first spike (inv_time_to_first_spike), 1.0 over the first interspike interval (inv_first_ISI), 1.0 over the second interspike interval (inv_second_ISI), 1.0 over the third interspike interval (inv_third_ISI), 1.0 over the fourth interspike interval (inv_fourth_ISI), 1.0 over the fifth interspike interval (inv_fifth_ISI), and 1.0 over the last interspike interval (inv_last_ISI).

#### Depolarization block test

HippoUnit implements the Depolarization Block Test as a series of somatic current stimulations. NEURON’s built-in single pulse current clamp point process is used for current injection. The current intensity is in the range of 0 nA to 1.6 nA with a step of 0.05 nA. Target features are the current threshold for the depolarization block (${I}_{th}$) and the membrane potential during the depolarization block (${V}_{eq}$). In addition, the test displays the I/O curve^7^.

#### Postsynaptic potential attenuation test

The excitatory postsynaptic potential (EPSP) is evoked by excitatory post-synaptic current (EPSC)-shaped current stimuli on the apical trunk at different distances from the soma. NEURON’s built-in synapse model Exp2Syn (τ_rise_ = 0.1 ms, τ_decay_ = 3 ms, EPSC_amp = 0.03 nA) is used to stimulate the dendrite. To set the synaptic weights, a stimulus with a weight of 0 is first used for each location. The last 10% of the evoked trace is averaged and the resting membrane potential (Vm) is determined. The synaptic weight required to induce EPSC with the experimentally determined amplitude (EPSC_amp) is calculated as: $weight=\frac{-EPSC\_amp}{Vm}$. For each selected location, the peak amplitude of local EPSP and somatic EPSP is measured, and the postsynaptic potential attenuation is calculated as $attenuation=\frac{{EPSP}_{soma}}{{EPSP}_{dend}}$. To calculate the error scores, the average values of the attenuation in the regions 100 µm, 200 µm, and 300 µm from the soma are first calculated. Feature Z-scores are calculated by comparing the average attenuation values and the observed data. The total score represents the average of the feature Z-scores^7^.

#### Backpropagating action potential (bAP) test

In the HippoUnit, the Backpropagating Action Potential Test is implemented in two steps. In the first step, the amplitude of the somatically injected current at which the cell fires at a frequency of around 15 Hz is found. In the next step, a current of this amplitude is injected into the soma. The amplitudes of the first and last AP of a train are measured at the distances of 50 µm, 150 µm, 250 µm and 350 µm from the soma^7^.

#### Oblique integration test

The Oblique Integration Test is implemented in the HippoUnit package in two steps. In the first step, appropriate dendrites are found. They must meet two conditions: (1) they must be near to the soma (distance < 200 µm), and (2) the synaptic input must not evoke a somatic AP. Then each selected dendrite is tested with an increasing number of synchronized and asynchronized inputs at the proximal and distal locations. One synaptic input is modeled as two components (AMPA and NMDA) with the same time of stimulus delivery. NEURON’s built-in synapse model Exp2Syn is used as an AMPA component. HippoUnit’s built-in synapse model with Jahr & Stevens voltage dependence is used as an NMDA component^7,26^. The synaptic weight of NMDA synapse is half the magnitude of the AMPA synapse. In the scenario of synchronous activity, 10 synaptic input activations are delivered to the tested dendrite. The interval between inputs is 0.1 ms. The asynchronous scenario is modeled with 10 synaptic inputs and 2 ms interval between inputs. Dendritic EPSPs at the place of stimuli and EPSPs at the soma are recorded during the test. The target features tested are mean threshold for dendritic spike initiation measured at the soma, proximal and distal threshold for dendritic spike initiation measured at the soma, degree of nonlinearity at the threshold, suprathreshold degree of nonlinearity, peak derivative of somatic voltage at the threshold, peak amplitude of somatic EPSP, time to peak of somatic EPSP and degree of nonlinearity in the case of asynchronous inputs^7^.

#### Run time measurement

We implemented a measurement of runtime of each test for each model. We started measuring time just before calling the main procedure that triggers the test. We ended the time measurement after the end of the given method. Thus, timing involves preparing the model for testing, testing itself, calculating scores, and saving the results. We ran each test on each model ten times and calculated the average test runtime. HippoUnit tests use the Pool class from the Python multiprocessing module for parallel simulations in one neuronal model, for example for stimuli of different intensities or stimuli at different locations on dendrites. The pool size was set to 10.

### Code availability

The models source code and Python scripts needed to run HippoUnit tests are available in the GitHub repository, https://github.com/tomko-neuron/HippoUnit/tree/master/paper. The source code of the CA1 model can be found in ModelDB, http://modeldb.yale.edu/266901.

## Results

### Somatic features of tested models

First, we assessed the ability of the five tested CA1 pyramidal cell models to simulate somatic electrophysiological features (one full-morphology M18 model and four reduced-morphology models: our To21 and published C10, CP15, Tu19 models, see Methods). For this purpose, ten 400 ms current injections with amplitudes in the range from − 1.0 nA to 1.0 nA were applied to each tested model. In CA1 pyramidal neurons, injection of hyperpolarizing current induces an I_h_-mediated voltage sag whereas injection of depolarizing current elicits firing with accommodation of the AP frequency^27^. The resting membrane potential (RMP) of a single CA1 pyramidal cell is between -64 mV^28^ and -84 mV^29^. These and other electrophysiological features were extracted from experimental voltage traces in the eFEL library (see Methods for details). With the exception of the CP15, a total 232 features were extracted and evaluated in all models. The CP15 model was weakly excitable, which means that at the current amplitude of 0.8 nA it fired only one spike and at the current amplitude of 1.0 nA it only fired three spikes. Therefore, it was not possible to extract all features.

As expected, the M18 model, which has been tuned for selected somatic features before^17^ and our newly tuned model, performed best in this test. The M18 model has reached errors lower than 1 in 58 features. Errors lower than 2 in all 61 features were reached by the M18 model and our model (Fig. 1d).

The voltage base is the average voltage during the last 10% of time before the stimulus^25^. The experimentally measured value of voltage base varies between -69.2 ± 4.5 mV (input current amplitude 0.2 nA) to -69.9 ± 4.6 mV (input current amplitude 1.0 nA)^17^. The models do not capture differences in voltage base across steps and therefore have fixed voltage base values corresponding to their resting potentials (Table S3): C10 (− 63.8 mV), CP15 (− 66.3 mV), Tu19 (− 65 mV), To21 (− 72.7 mV), and M18 (− 69.6 mV). The M18 model was the best in this feature with an average error of 0.0459. The remaining models displayed average errors below the maximum error accepted.

The voltage deflection reflects a hyperpolarized state that is revealed at negative input currents^25^. An average error lower than 1 was reached by models of CP15, To21, and M18. The remaining models had an average error greater than 2 (Fig. 1b).

The spike count reflects the number of spikes fired during the stimulus^25^. Figure 1c plots the spike numbers against the injected current for all tested models together with experimental data. The C10 and Tu19 models were too excitable, resulting in a high average error. (Fig. 1c, d). The experimental data were fitted best by the M18 model (Fig. 1c), as confirmed by the lowest average error (Fig. 1d). CP15 and To21 models also achieved relatively low errors of 1.4 and 1, respectively.

Time to the first (or last) spike is the time from the start of stimulus to the maximum of the first (or last) peak^25^. These were measured for currents of 0.8 nA and 1.0 nA. All models produced realistic spike times with an error of less than 2 (Fig. 1d). Time to the first spike was also expressed as the inverse value – inv_time_to_first_spike, calculated as 1 over time to the first spike. Models of CP15, To21, and M18 achieved score less than 2 and models of C10 and Tu19 achieved scores greater than 2.

Other spike event features include inverted interspike intervals, namely inv_first_ISI, inv_second_ISI, inv_third_ISI, inv_fourth_ISI, inv_fifth_ISI and inv_last_ISI. The error of the first ISI was less than 2 in all models. For the remaining ISI intervals, errors of models of CP15, To21, and M18 were still below 2. However, errors of models of C10 and Tu19 increased with each next interspike interval.

The final score of this test for each model is compared in Fig. 6. The CP15 model’s performance was surprisingly good considering the fact that it was not tuned for this test. The M18 and To21 performed best.

### Depolarization block test

**Figure 2:**
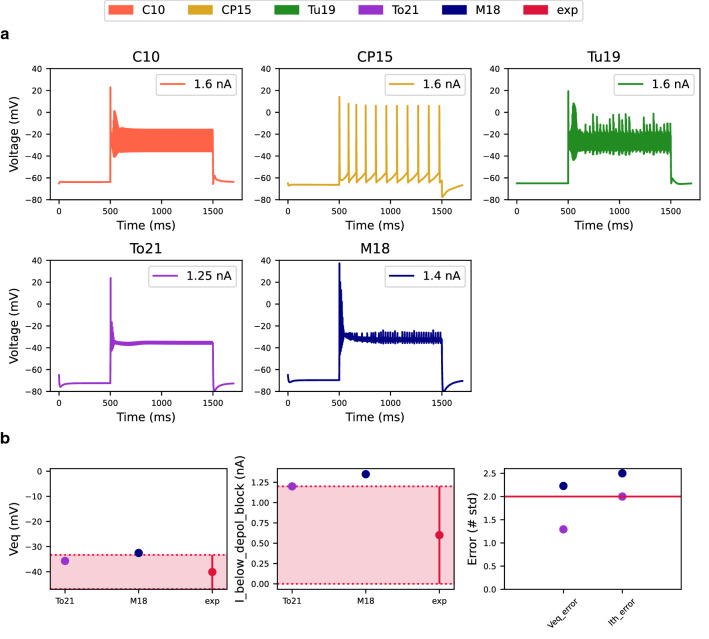
The Depolarization Block Test expressed the ability of models to enter depolarization block under sustained input current. (**a**) Somatic voltage response of models to the current at which the models entered depolarization block. If the model did not enter depolarization block, the somatic voltage response is the response to the maximum current intensity. (**b**) The values of the equilibrium voltage during depolarization block, the current intensity before the model enters depolarization block, and associated errors in these features expressed by the number of standard deviations for models that have entered depolarization block. Experimental values with the double standard deviations were obtained from the paper of Bianchi et al.^12^. The red horizontal line represents $sd=2$ and determines the maximum error accepted.

Next, we tested the readiness of the models to enter depolarization block. For this we performed several somatic current injections for each tested model. We started with an initial amplitude of 0.05 nA and gradually increased it by 0.05 nA up to a final amplitude of 1.6 nA. We measured the current threshold needed for the models to enter depolarization block. Below a threshold value, the models fire a regular, weakly adapting, train of APs for the entire duration of the current step. If the models are capable of entering depolarization block (Fig. 2a), above the threshold value and during the current step, the AP amplitude decreases to zero and the membrane potential reaches an equilibrium point (Veq)^12^.

Only M18 and To21 models entered depolarization block. For models that did not enter depolarization block, we plotted the somatic response to the highest current intensity of 1.6 nA (Fig. 2a). The equilibrium (steady state) value of the membrane potential (Veq) during the depolarization block was experimentally determined to be − 40.1 ± 3.4 mV^12^. For the To21 the value of Veq was = − 35.9 mV, which corresponds to an error of 1.2. The M18 displayed slightly more depolarized equilibrium values (Veq = − 32.52 mV, error = 2.2) (Fig. 2b).

Both models had a higher current threshold for entering depolarization block than the experimental value of 0.6 ± 0.3 nA^12^. For the To21 model it was 1.2 nA (error = 1.8) and for the M18 model it was 1.4 nA (error = 2.5) (Fig. 2b). The final score in this test was calculated only for the models that entered depolarization block. The To21 model achieved a score of 1.6 and the M18 model reached a score of 2.4 (Fig. 6).

### Postsynaptic potential attenuation test

To test the models for their ability to replicate the attenuation of EPSPs, we applied several stimuli using a double exponential synapse at various distances from the soma on the apical trunk. At the same time, we recorded the amplitudes of the EPSP at the stimulus site and at the soma. Finally, we calculated EPSP attenuation value as EPSP_soma_/EPSP_dend_ for a given distance. The attenuation value expresses the ratio between the EPSP amplitude evoked at the dendrite and the EPSP amplitude measured at the soma, quantifying how much of the dendritically evoked EPSP amplitude is retained at the soma. For model validation, we calculated the errors between model EPSP attenuation values and experimental EPSP attenuation values at a distance of about 100 µm, 200 µm, and 300 µm from the soma.

**Figure 3:**
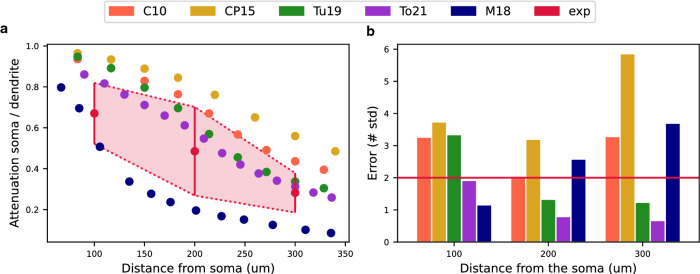
The Postsynaptic Potential Attenuation Test evaluated the rate of attenuation of excitatory postsynaptic potentials (EPSPs) during their propagation along the apical trunk of the tested models. (**a**) The rate of attenuation of postsynaptic potential calculated as EPSP_soma_/EPSP_dend_ at different distances from the soma on the apical trunk. Experimental values with the double standard deviations are obtained from the paper of Magee and Cook^30^. (**b**) Model errors in the EPSP attenuation expressed by the number of standard deviations at different distances from the soma. The red horizontal line represents $sd=2$ and determines the maximum error accepted.

For all tested models, the postsynaptic EPSP attenuation was enhanced with increasing distance from the soma. This was reflected in the decreasing EPSP_soma_/EPSP_dend_ ratio with increasing distance from the soma (Fig. 3a). These simulations were qualitatively consistent with the experimental data from Magee and Cook^30^.

In general, the M18 model showed the strongest attenuation. In contrast, the CP15 model showed the weakest attenuation (Fig. 3a). We compared the attenuation errors of the models at different distances from the soma. At the distance of 100 µm, the models that had acceptable error less than 2 were the M18 (error = 1.15) and our model (error = 1.92). In the middle of the apical trunk, at 200 µm from the soma, the To21 and the Tu19 models had the smallest error (1.08 and 1.86 respectively). Also, at the distance of 300 µm from the soma, the two latter models had the lowest error (1.07 and 1.82, respectively) (Fig. 3b). The final score of this test for each model is compared in Fig. 6.

### Backpropagating action potential (bAP) test

Next, we compared the models with respect to the attenuation of bAPs, which represent plasticity-relevant depolarization events. In CA1 pyramidal cells, the AP is typically initiated in the axon hillock and propagated forward to the axon and backward to the dendritic tree as a bAP. The bAP amplitude attenuates with increasing distance from the soma. At a distance of 280 µm from the soma, the attenuation is less than 50%. However, at a distance beyond 300 µm from the soma, there is a dichotomy of bAP spreading, with either strong bAP attenuation of 70–85% (i.e. weak backpropagation) or weak bAP attenuation of 25–45% (i.e. strong backpropagation), respectively^24^. This dichotomy was observed only for the first spike in a train of APs, whereas the attenuation of the last spike was similar to the attenuation of the first spike in weakly-propagating cells. This can be explained by the difference in the degree of amplification of the AP by voltage-gated channels in the distal dendrites^24^.

**Figure 4:**
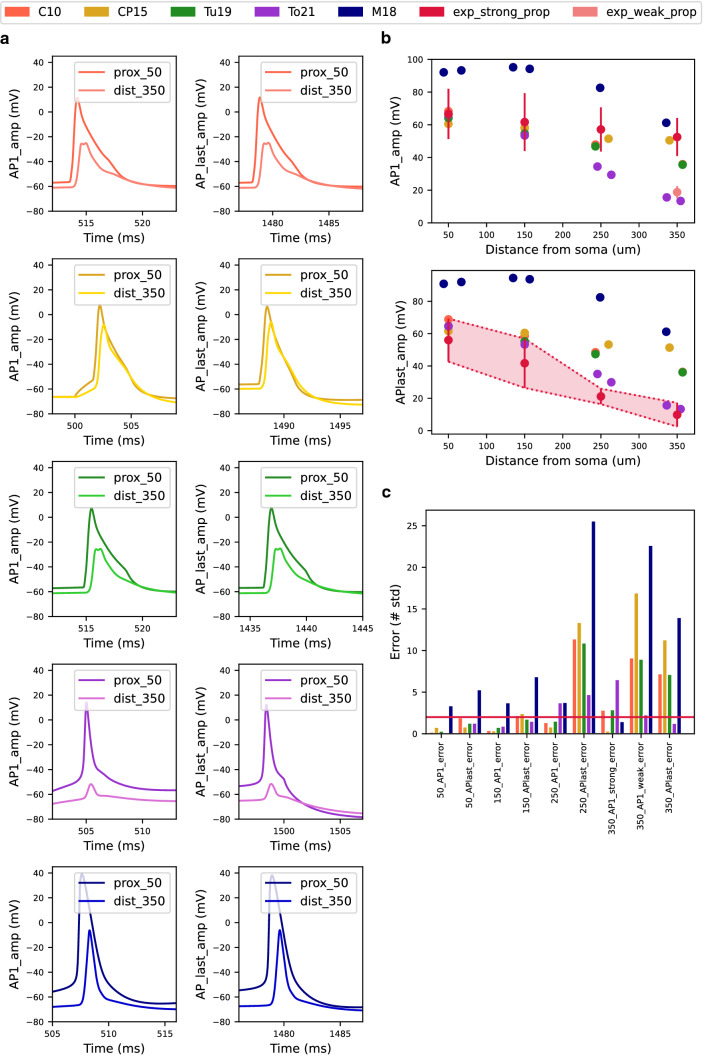
The Backpropagating Action Potential (bAP) Test evaluates the models' ability to reproduce experimentally determined bAP attenuation. (**a**) The first and last AP from the AP train recorded at distances of 50 µm and 350 µm from the soma. (**b**) The amplitude of the first AP (top) and the amplitude of the last AP (bottom) from the AP train decreased with increasing distance from the soma. Experimental values with the double standard deviations for bAPs are obtained from measurements of Golding et al.^24^. Based on the first bAP, CA1 pyramidal cells can be divided into two groups – weakly-propagating and strongly-propagating (top)^24^. (**c**) Model errors in the backpropagation of APs expressed by the number of standard deviations at different distances from the soma. The red horizontal line represents $sd=2$ and determines the maximum error accepted.

In the tested models we compared the effectiveness and the mode of backpropagation of APs. To do this, the bAP Test uses somatic current injection to generate a train of APs at a frequency of about 15 Hz and measures the amplitudes of the first AP and the last AP in the train at various positions on the apical trunk. However, the CP15 model did not reach a firing frequency of about 15 Hz and thus the test failed. For this reason, we wrote our own implementation of the bAP test for this model. We plotted the voltage trace of the first and last AP at the proximal and distal locations on the apical trunk (Fig. 4a). It is clear from these figures that our model is weakly-propagating and the remaining models are strongly-propagating.

Here, however, it is important to note that in all tested simplified models, the distal 200 µm of the apical trunk are modeled as a single NEURON section with a low number of compartments and therefore larger bAP-related errors may occur in this region due to the low spatial resolution. This should be taken into account when interpreting the bAP test results.

In the case of the first AP, the C10, CP15, Tu19, and To21 models obtained similar amplitude values in the proximal parts of the apical trunk (< 200 µm) as compared to experimental data. In the distal part of the apical trunk, C10, CP15, and Tu19 models reproduced qualitatively the bAP values of strongly-propagating cells. In contrast, our model reproduced the bAP values of weakly-propagating cells. At most distances from soma, M18 model generated bAPs with markedly higher amplitudes than observed in the experiments (Fig. 4b). At a distance of 350 µm from the soma, it reached similar values to those measured in the experiment (Fig. 4b). Based on the weak bAP attenuation, we can classify it as a strongly-propagating model. Based on the results, we can conclude that the CP15 model best captured the observed data for the first AP in strongly-propagating cells.

The amplitude of the last AP of the spike train diminished in all models as the distance from the soma increased. Experimental data were best fit by our model. For the C10, CP15, and Tu19 models, the backpropagation was slightly stronger than experimental values. Similarly to the first amplitude, the M18 model had higher values of the last AP in the AP train than the observed data in all parts of the apical trunk (Fig. 4b).

Model errors for the first and last AP amplitudes at each measurement site on the apical trunk are shown as a bar plot (Fig. 4c). The graph shows that errors greater than 2 occurred predominantly in bAP measurements in the distal parts of the apical trunk. One of the factors that could have caused the strong backpropagation was that only one longer NEURON section with a relatively low spatial resolution (i.e. with a low number of compartments) was used to model this part of the apical trunk in published reduced models, but not in our model.

In calculating the final test score, we took into account that the bAP in CA1 cells was propagating in two modes. Therefore, each model had a calculated score for strong and weak bAP propagation data, respectively. In this test our model performed best. The total score of the models is shown in Fig. 6.

### Oblique integration test

For testing the dendritic integration properties of CA1 models, we used the Oblique Integration Test. The test was applied to three models, namely Tu19, To21 and M18. The C10 model was not tested because this model had no explicitly modelled oblique dendrites. The CP15 model was also not tested because no appropriate dendrites which met the two conditions for testing (see Methods) were found in this model.

**Figure 5:**
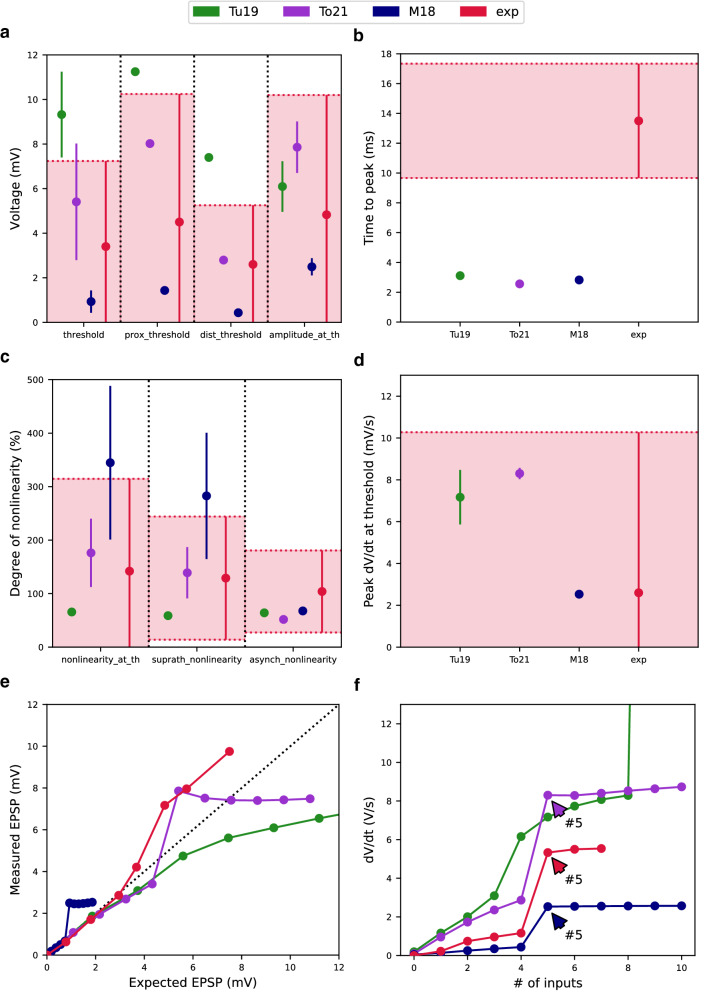
The Oblique Integration Test evaluated nonlinear dendritic integration of asynchronous and synchronous synaptic inputs in the oblique dendrites of the tested models. (**a**) The mean threshold for dendritic spike initiation, the proximal threshold for dendritic spike initiation, and the distal threshold for dendritic spike initiation, and the amplitude at the threshold measured at the soma with a dendritic spike present. (**b**) The increase in the somatic voltage upon a dendritic spike can be characterized by the time in which the maximum amplitude is reached (time to peak). All models had this value significantly lower than experimental data. (**c**) The mean threshold nonlinearity associated with a dendritic spike, suprathreshold nonlinearity and the degree of nonlinearity for asynchronous input patterns calculated for each tested model. (**d**) The peak temporal derivative of somatic voltage response at the threshold for dendritic spike initiation. (**e**) Averaged input – output curves for synchronous inputs of all tested models. A sharp increase in the curve indicates a dendritic spike. Dashed line represents linear summation. (**f**) Plot of dV/dt versus number of inputs. Arrows indicate the number of synchronous inputs required to induce a dendritic spike. Note a sharp increase in the somatic voltage during the dendritic spike initiation. All experimental values with the double standard deviations were obtained from the paper of Losonczy and Magee^31^.

To investigate how tested dendrites integrate incoming synaptic inputs, we measured dendritic EPSPs at the stimulation location. Evoked EPSPs could be detected as an increase in the somatic voltage and for this reason we also measured the somatic response to synaptic stimulation. EPSPs evoked by an asynchronous input pattern (2 ms interval between inputs) were summed in a sublinear manner in all models. However, the Tu19 model generated a dendritic spike when a stimulation pattern consisted of 10 inputs (Fig. 5f). In the case of a synchronous input pattern (0.2 ms interval between inputs), all models summed EPSPs supralinearly, resulting in the generation of a dendritic spike (Fig. 5e). In the case of the M18 and To21 models, five synchronous inputs were required to evoke a dendritic spike. In the case of the Tu19 model, up to nine synchronous inputs were required. From the experimental data it is known that higher levels of synchronous input (five to seven spines at 0.1 ms interval) lead to dendritic spike generation^31^.

Dendritic spikes were detected at the soma as a sharp increase in the somatic voltage. This increase in the somatic voltage consisted of a fast and a slow phase. We calculated the peak temporal derivative of the somatic voltage (dV/dt) for each number of inputs. Based on this, we could determine the voltage threshold for generating a dendritic spike. We determined the threshold separately for proximal and distal locations and from these values we calculated a mean threshold (Fig. 5a, 1st—3rd columns). The peak (dV/dt) values at a threshold for each model are shown in Fig. 5d. The amplitude at the threshold (Fig. 1a 4th column) was calculated as a mean of measured EPSP amplitudes at the threshold for each tested location. We characterized a slow phase of the somatic voltage increase using a mean time to peak, during which somatic EPSP reached its peak amplitude (Fig. 5b). All tested models had a markedly lower values than observed in experiments.

To quantify the degree of nonlinearity, we calculated the mean nonlinearity at the threshold, the suprathreshold nonlinearity and the asynchronous nonlinearity using formulas in Losonczy and Magee^31^. The M18 and To21 models reached higher mean degree of nonlinearity at the threshold and the suprathreshold nonlinearity than experimental values but mean values of our model remained in the experimental range (Fig. 5c). The Tu19 model had values slightly below 100%. All models were in the experimental range for the asynchronous nonlinearity (Fig. 5c).

**Figure 6:**
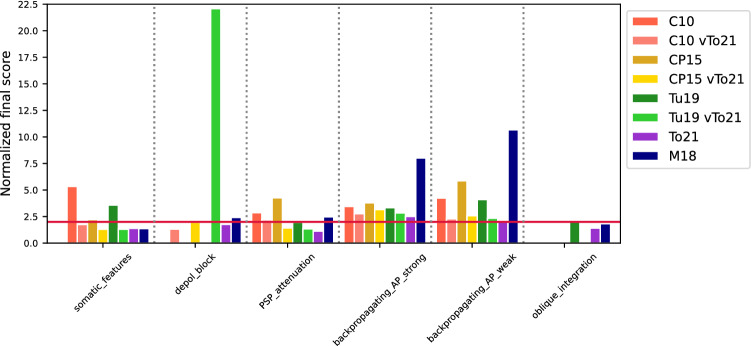
The final error score of all models achieved for each test. The final error score for a given test is expressed as the average of the errors of all tested features (see Methods). If the model does not pass the test, it is indicated by a blank space. The figure also contains the final scores from the modified reduced-morphology models labeled as "vTo21" at the end of the model name in the legend (see text for more details). The red horizontal line represents *sd* $=2$.

In terms of the overall score, the To21 and M18 models scored below 2. The Tu19 model achieved an overall score of 2.14 in this test (Fig. 6). Figure 6 shows all final scores of the models achieved in each test as the number of standard deviations. We can see that our CA1 pyramidal cell model reached the average lowest error when taking into account all of the tests.

### Model runtimes and the importance of biophysical mechanisms for the performance of reduced-morphology models

**Figure 7:**
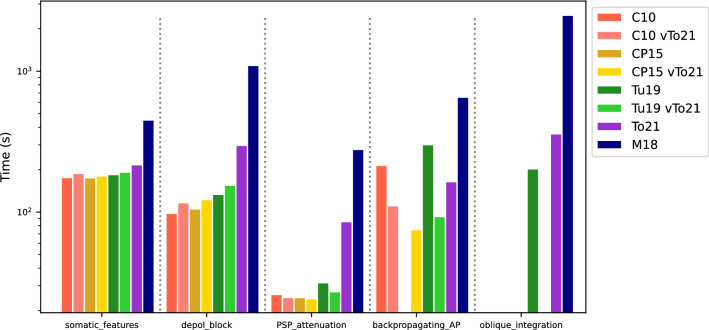
The runtimes of all models for each test. If the model does not pass the test, it is indicated by a blank space. The figure also contains the runtimes of the modified reduced-morphology models, which is indicated as "vTo21" at the end of the model name in the legend (see text for more details). The bAP Test for the CP15 model was implemented by us and therefore we did not measure the runtime.

To estimate the computational speed of the models, we measured runtimes for each model and each test. The runtimes are shown in Fig. 7. As expected, testing took the longest for the M18 model, as it is a full-morphology model. It contains the largest number of compartments for which calculations need to be performed. Testing on reduced models was 2–7 times faster, depending on the test performed. In the case of network models, this time saving could be even higher. The most significant factor in reducing the runtimes is the smaller number of compartments as compared to the full-morphology M18 model.

In the case of reduced models, the number of compartments and branch points is similar. The C10, CP15, Tu19 models have similar ion channel content whereas the To21 model has similar channels to the M18 model. Both groups have similar channel types with some exceptions: (1) The K_M_ channel in the To21 and M18 models is adapted for CA1 cells^32^ and plays an important role in the cell's entry into the depolarization block (see Discussion). (2) The C10, CP15, Tu19 models contain Ca_L_, Ca_R_, Ca_T_ calcium channels and To21 and M18 contain Ca_L_, Ca_N_, Ca_T_ calcium channels. The difference is also in the implementation of the calcium accumulation mechanism. (3) The C10, CP15, Tu19 models have a complete channel content inserted only in the soma and in a subset of sections forming the apical trunk and basal dendrites. On the other hand, the To21 and M18 models have a complete set of channels in each section. Detailed information is given in Table S3.

It follows from the above that the C10, CP15 and Tu19 models should have similar runtimes. This was confirmed by our runtime measurements (Fig. 7). The runtimes for the To21 model were higher as compared to the other reduced-morphology models, which could be due to the differences mentioned in the point (3) in the previous paragraph. In line with this, a slight increase in runtimes for modified reduced-morphology models (vTo21) may be explained by the insertion of all channels into all sections in these models. In the case of the bAP test, a significant proportion of the total runtime is the first step of the test, which is to find a suitable current intensity at which the model fires around 15 Hz. This search can significantly extend the total runtime. Examples are the Tu19 and Tu19 vTo21 models, where twice as many simulations (IClamps) were required for the Tu19 model as for the Tu19 vTo21 model.

Importantly, the final error scores of the modified reduced-morphology models in the tests improved markedly (Fig. 6). These results show, first, that ion channel biophysics is the key property important for the successful performance in the standardized HippoUnit tests and, second, that the performance of available reduced-morphology models can be easily improved by implementing biophysics from the realistic (To21 and M18) models.

## Discussion

The major aims achieved in our work can be summarized as follows: We introduce a new biophysically realistic reduced-morphology model of the CA1 pyramidal cell. Due to its realistic biophysics, our model preserves canonical electrophysiological features defined by HippoUnit while decreasing computational time due to its low morphological complexity. The model successfully reproduces somatic, dendritic, and synaptic electrophysiological features of CA1 pyramidal neurons. These include somatic voltage and spiking responses, bAP and EPSP attenuation as well as nonlinear synaptic integration triggering dendritic spikes. The performance of the model was similar to a published full-morphology model that had been previously tuned to simulate some of these features^17^. When comparing the final error score for all the tests combined, our model performed better than the three other reduced-morphology models, and even slightly better than the full-morphology model. The benefit of our validated model for the neuroscientific community is that it can be used as a replacement for full-morphology models for faster simulations of single cell behavior. Furthermore, although we did not test it explicitly, we expect it to help significantly reduce runtimes for network simulations.

The HippoUnit test suite is a Python-based tool for systematic testing of CA1 pyramidal cells models. So far, this tool has only been used to test complex full-morphology models^7^. Here, for the first time we used it to test simplified reduced-morphology models. For a comparison with a full-morphology model, we included also the M18 model. The reason for this was that it has been previously tuned for some of the HippoUnit features and therefore it can be viewed as a reference model. Moreover, its biophysics provided the basis for active mechanisms (including their parameters) implemented in our model.

The better performance of our model as compared to the three other reduced-morphology models is not surprising, as they were created to capture specific CA1 pyramidal cell behavior (see the Introduction and Methods) and not tuned for HippoUnit tests. Importantly, our purpose was not to criticize the validity of these models in biology but rather to use them as controls for the improvement and validation of our model. The models remain valid for the cell behavior they were tuned for. Moreover, by changing their biophysical mechanisms (see Figs. 6, 7), we showed that they can be easily adjusted to perform well in the HippoUnit tests (see the “vTo21” models in Results). Thus, using our or similar approaches, future users of reduced-morphology ModelDB models can extend their validity beyond phenomena they were tuned for by adapting them with the standardized HippoUnit tests.

### Somatic voltage response

The Somatic Features Test revealed that the M18 model reached the best score. This was not unexpected, as this model has been previously optimized for some of the somatic features^17^. The results show that the C10 and Tu19 models are highly excitable, which is manifested by high spike counts, firing rates and low interspike intervals. In contrast, the CP15 model was weakly excitable. The somatic properties of the models depend strongly on their perisomatic ion channel content (see Table S3). The models differed in their maximum channel conductance values and this accounts for their different test performance. This interpretation is supported also by the observation that inserting M18/To21 ion channels into the three untuned reduced-morphology models significantly decreased their error score for somatic features (see the “vTo21” models in Figs. 6, 7).

### Depolarization block

CA1 pyramidal neurons respond to a strong current injection with a depolarization block. When the current intensity exceeds a certain threshold, neurons stop firing while maintaining the membrane potential at a constant level throughout the stimulation period. Sodium (*NaT*) and potassium (*K*_*DR*_) channels, which underlie the basic spiking mechanism, play a major role in this response of neurons. In particular, the interaction between their activating and inactivating properties is of key importance^12,33^. The depolarization block emerges when, during an interval between subsequent APs, the *NaT* channel does not deinactivate completely and *K*_*DR*_ does not activate sufficiently to repolarize the neuron. Both conditions are met in I_Na_ and I_KDR_ models by Shah et al.^32^ that were used also in the benchmark M18 model and our To21 model. Whereas *NaT* and *K*_*DR*_ are sufficient for the depolarization block, *K*_*M*_ and *K*_*mAHP*_ channels determine the equilibrium voltage during the block^12^. Previous simulations have shown that the steady-state activation and inactivation of I_Na_ and I_KDR_ currents from Poirazi et al.^10,11^ prevents reaching depolarization block^12^. This is relevant since the C10, CP15, and Tu19 models use the I_Na_ and I_KDR_ models from Poirazi et al.^10,11^. Taken together, this explains why the M18 and To21 models successfully enter depolarization block whereas the untuned control models do not. The To21 model reached the best score because we adjusted the maximal conductance of I_Na_, I_KDR_, I_KM_, I_CaL_, and I_CaT_ to the same values as reported in Table 1 in Bianchi et al.^12^ who focused on reproducing the depolarization block.

### Postsynaptic potential attenuation

CA1 pyramidal cells display a characteristic strong dendrosomatic attenuation of excitatory postsynaptic potentials^30,34^. Local injections of EPSC-shaped currents into different sites of the dendritic tree induce location-dependent EPSPs. As the distance between the stimulated site and the soma increases, the amplitude of these locally evoked EPSPs increases, but at the same time, the EPSP amplitudes recorded at the soma decrease^30^. However, for Schaffer collateral inputs, the amplitude of synaptically evoked somatic EPSPs is independent of synapse location^30^ due to a distance-dependent increase in postsynaptic AMPA conductance and AMPA receptor content^35,36^.

Such experimentally observed EPSP attenuation was better captured by our model than by the reference M18 model or the three other reduced-morphology models. In our model, the recorded peaks of the amplitudes of local and somatic EPSPs were similar to those in figure 2B in Magee and Cook^30^. In the case of the M18 model, the local EPSPs were larger and their dendrosomatic attenuation was stronger than indicated by the range of experimental values. Also, the remaining C10 and CP15 models captured the EPSP attenuation only qualitatively. Only the Tu19 model showed attenuation values similar to the experimental data. Overall, the dendritic filtering was too strong in the reference full-morphology M18 model and too weak in the C10 and CP15 models. Thus, the interplay between dendritic geometry and biophysical cable properties (especially the axial and membrane resistivity, and the distribution of I_h_, (see Golding et al.^34^) would require further fine tuning in these models to reproduce the EPSP attenuation data not only qualitatively but also quantitatively.

### Backpropagation of action potentials

APs backpropagate into the apical trunk and basal dendrites of the CA1 pyramidal cell becoming weaker with increasing distance from the soma. Regarding the backpropagation of APs, the CA1 pyramidal cells can be distinguished as strongly- and weakly-propagating. When comparing the bAP amplitude in the proximal and distal parts of the apical trunk, strongly-propagating cells lose approximately 25–45% of the AP amplitude, while weakly-propagating cells lose up to 70–85%^24^. Different distributions of sodium and A-type potassium channels are sufficient to account for the bAP dichotomy^24^. In agreement with this, the maximum conductance of the I_KA_ had an increasing gradient in the C10, CP15, and Tu19 models with increasing distance from the soma, leading to a regime between weak and strong backpropagation. An adjustment of the conductances would move these models closer to weak or strong backpropagation experimental results. The relatively high error of the benchmark M18 model was due to high somatic AP amplitude caused by strong axonal sodium conductance. In contrast, our model used a slightly reduced sodium conductance. The result was a somatic AP with similar characteristics to those in Golding et al.^24^ (see their figure 3). At the same time, following Golding et al.^24^, we used an increasing gradient of I_KA_ in the apical trunk which resulted in weak AP backpropagation. In summary, the good performance of our model in the bAP Test can be explained by the fine-tuning of I_Na_ and I_KA_ based on bAP simulations and experiments of Golding et al.^24^.

### Synaptic integration in dendrites

Most excitatory synaptic inputs of CA1 pyramidal cells are located on thin radial oblique dendrites in the stratum radiatum. Signals from these inputs are integrated linearly or supralinearly depending on the degree of their synchronization. Asynchronous inputs exhibit linear summation while synchronous inputs are summed supralinearly, both in the case of clustered and distributed configurations^13,31^. The result of such supralinear summation is a dendritic spike. This supports the idea that a single radial oblique dendrite of the CA1 pyramidal cell serves as a single integrative compartment or subunit^10,31^.

The Oblique Integration Test evaluated how the models integrated synchronous and asynchronous synaptic inputs. Since synapses had to be placed on oblique branches, the test was run only on three models, M18, Tu19, and our model. In the case of synchronized inputs, dendritic spikes were generated in line with data. It is known that at least five synchronized inputs are required to generate a dendritic spike^13,31^. In agreement with these experiments, such low number of activated inputs was needed for the M18 and To21 models. The Tu19 model required nine synchronous inputs to generate the dendritic spike. A putative dendritic spike can be detected on the soma as a rapid increase in the somatic voltage^31^. We saw a similar increase in all the models. In addition, we observed that the level of non-linearity was in the experimental range for all models. On the other hand, asynchronous inputs are known to sum up linearly at the lateral branches of CA1 neurons^31^. In the three models, the synaptic integration of asynchronous inputs was slightly sublinear but still in the experimental range. The time to peak for corresponding somatic events was faster than in experiments and would require tuning of dendritic filtering or possibly implementing synaptic inhibition. Taken together, the models were able to generate realistic synaptically driven dendritic spikes. These results indicate that the presence of dendritic sodium and calcium channels are sufficient for mimicking nonlinear integration of dendritic synaptic events.

### Limitations of the model

In our model, we used a set of channels from the M18 model. Subsequently, we manually adjusted the conductances of sodium, potassium, and calcium channels. However, neurons exhibit high (~ 2 - 6-fold) variability of ion currents and ion channel expression^37–40^. Computational modeling suggests that this is the case also for CA1 pyramidal cells^17^. Therefore, it is important to emphasize that the final ion channel composition of our model represents only one possible realistic solution in the parameter space. In the future, we would like to use population-based (also called ensemble or database) modeling^41–45^ to generate populations of realistic reduced-morphology models that would reflect the variability of experimental data. For this purpose, an update for the distribution of CA1 pyramidal cell ion channels based on available literature would be desirable as recently done for hippocampal dentate granule cells^6^.

## Supplementary Information

Supplementary Information

## Data Availability

The datasets generated during and analyzed during the current study are available in the GitHub repository, https://github.com/tomko-neuron/HippoUnit.
