# Supplementary material for: A new reduced-morphology model for CA1 pyramidal cells and its validation and comparison with other models using HippoUnit
Source: Sci Rep. 2021 Apr 7;11:7615. doi: 10.1038/s41598-021-87002-7 (PMC8027802; doi:10.1038/s41598-021-87002-7)
Supplement: Supplementary file 1 — Supplementary Information [file 41598_2021_87002_MOESM1_ESM.pdf]

Supplemental information

A new reduced-morphology model for CA1 pyramidal cells and its validation and comparison with other models using HippoUnit

Matus Tomko, Lubica Benuskova, Peter Jedlicka

Supplementary Table S1: Uniformly distributed passive parameters, active ionic conductances and reversal potentials of channels for each section of the To21 model

Mechanism	Somatic	Axonal	Basal	Apical	Trunk
C_m (μF/cm^2)	1	1	1	1	1
R_A (Ω/cm^2)	115.3958	85.2024	115.3958	115.3958	115.3958
g_{Na} (S/cm^2)	0.035	0.035	0.03828	0.03828	0.025
g_{Kdr} (S/cm^2)	0.0015	0.01166	0.004304	0.004304	0.02
g_{KM} (S/cm^2)	0.001	0.02647	-	-	-
g_{KCa} (S/cm^2)	0.0015	-	9.0311e-05	9.0311e-05	9.0311e-05
g_{KCagk} (S/cm^2)	4.4820e-05	-	4.4820e-05	4.4820e-05	4.4820e-05
g_{CaL} (S/cm^2)	0.0005	-	8.0325e-06	8.0325e-06	8.0325e-06
g_{CaN} (S/cm^2)	2.2619e-06	-	2.2619e-06	2.2619e-06	2.2619e-06
g_{CaT} (S/cm^2)	0.00005	-	1.1849e-06	1.1849e-06	1.1849e-06
g_L (S/cm^2)	9.03139e-05	0.0001289	9.03139e-05	9.03139e-05	9.03139e-05
$[Ca^{2+}]_o$ (mM)	50e-6	-	50e-6	50e-6	50e-6
τ_{Ca} (ms)	100	-	100	100	100
E_{Na} (mV)	50	50	50	50	50
E_K (mV)	-90	-90	-90	-90	-90

Supplementary Table S2: K_A, I_h conductances and reversal potential of passive current in individual sections of the To21

Section / Mechanism	g_{KA} (S/cm^2)	g_{Ih} (S/cm^2)	E_L (mV)
Soma	0.0075	1.9042e-05	-65.7269
Axon	0.1637	-	-79.9171
Proximal SO dendrite	0.001433	5.0462e-05	-67.5602
Distal SO dendrite	0.02522	0.0001361	-72.5602
Thick proximal SR dendrite	0.1	5.04624e-05	-67.5602
Thick medium SR dendrite	0.15	0.0001076	-70.8936
Thick distal SR dendrite	0.2	0.0001933	-75.8936
Thin SR dendrite 1	0.006259	9.3308e-05	-70.06023
Thin SR dendrite 1	0.03834	0.0001504	-73.3936
Thin SR dendrite 1	0.1613	0.0002361	-78.3936
Thick SLM dendrite	0.1855	0.0002789	-80.8936
Medium SLM dendrite	0.1926	0.0003361	-84.2269
Thin SLM dendrite	0.1936	0.0003789	-86.7269

Supplementary Table S3: Active and passive properties used in models

Channel / Current name	Mod file	Sections				
		C10	CP15	Tu19	To21	M18
Leak conductance	pas	soma, rad, lm, ori, axon	soma, rad, lm, ori, axon	soma, rad, lm, ori, axon	soma, rad, lm, ori, axon	soma, rad, lm, ori, axon
HH mechanism	hha2	soma, axon	soma, axon	soma, axon		
HH mechanism	hha_old	rad, lm, ori	rad, obl, lm, ori	rad, obl, lm, ori		
H current	h	soma, rad, ori		soma, rad, ori		
H current	hd				soma, rad, obl, lm, ori	soma, rad, obl, lm, ori
H current	hd2		soma, rad, obl, ori			
Na^+ current for axon	nax				soma, rad, lm, ori, axon	soma, rad, lm, ori, axon
Proximal A-type K^+ current	kap	soma, rad, ori	soma, rad, obl,ori	soma, rad, ori	soma, axon	soma, axon
A-type K^+ current	kad	rad, lm, ori	rad, obl, lm, ori	rad, obl, lm, ori	rad, obl, lm, ori	rad, obl, lm, ori
Delayed rectifier K^+ current	kdr				soma, rad, lm, ori, axon	soma, rad, lm, ori, axon
M-type K^+ current	km	soma, rad, ori, axon	soma, rad, obl, ori, axon	soma, rad, ori, axon		
M-type K^+ current	kmb				soma, axon	soma, axon
Ca^{2+}-activated sAHP K^+ current	kca	soma, rad, ori	soma, rad, obl, ori	soma, rad, ori	soma, rad, obl, lm, ori	soma, rad, obl, lm, ori

Ca^{2+}-activated mAHP K^+ current	mykca	soma, rad, ori		soma, rad, ori		
Ca^{2+}-activated K^+ current	cagk				soma, rad, obl, lm, ori	soma, rad, obl, lm, ori
Ca^{2+}-activated K^+ current	cagk2		soma, rad, obl, ori			
HVA L-type Ca^{2+} current	cal	soma	soma	soma	soma, rad, obl, lm, ori	soma, rad, obl, lm, ori
HVA L-type Ca^{2+} current	calH	rad, ori	rad, obl, ori	rad, ori		
HVA N-type Ca^{2+} current	can				soma, rad, obl, lm, ori	soma, rad, obl, lm, ori
MVA R-type Ca^{2+} current	somacar	soma	soma	soma		
MVA R-type Ca^{2+} current	car	rad, ori	rad, obl, ori	rad, ori		
LVA T-type Ca^{2+} current	cat	soma, rad, ori	soma, rad, obl, ori	soma, rad, ori	soma, rad, obl, lm, ori	soma, rad, obl, lm, ori
Calcium pump	cad	soma, rad, ori	soma, rad, obl, ori	soma, rad, ori		
Calcium accumulation	cacum				soma, rad, obl, lm, ori	soma, rad, obl, lm, ori

Supplementary S4: Somatic Features Test detailed results

minimum voltage													
**Step (nA)**	**Experiment mean**	**Experiment std**	**C10 value**	**C10 error**	**CP15 value**	**CP15 error**	**Tu19 value**	**Tu19 error**	**To21 value**	**To21 error**	**M18 value**	**M18 error**
-1	-99.9313	9.7377	-143.502	4.474481	-114.562	1.502482	-139.077	4.020009	-116.241	1.674941	-107.226	0.749093
-0.8	-94.3532	8.1946	-129.832	4.329578	-103.146	1.07296	-125.937	3.854259	-107.22	1.570129	-99.8416	0.669758
-0.6	-88.5859	6.8969	-114.919	3.818052	-91.5606	0.431303	-111.759	3.359989	-98.1312	1.384	-92.372	0.548952
-0.4	-85.2382	9.5815	-98.8119	1.416657	-81.1776	0.423797	-96.6202	1.187912	-89.1218	0.405319	-84.8206	0.043588
-0.2	-77.8483	6.489	-81.5999	0.578153	-72.9466	0.755387	-80.5927	0.42293	-80.4264	0.397309	-77.212	0.098053

sag_amplitude												
**Step (nA)**	**Experiment mean**	**Experiment std**	**C10 value**	**C10 error**	**CP15 value**	**CP15 error**	**Tu19 value**	**Tu19 error**	**To21 value**	**To21 error**	**M18 value**	**M18 error**
-1	6.5995	3.3034	29.70236	6.99366	19.83881	4.007783	24.84155	5.522205	10.50972	1.183697	8.411166	0.548425
-0.8	5.2681	2.3817	26.86898	9.069521	16.12122	4.55688	22.8738	7.392074	8.832507	1.496581	6.887593	0.679973
-0.6	4.159	1.8053	21.3321	9.512601	10.83257	3.696655	18.21476	7.785829	6.662081	1.386518	5.180959	0.566088
-0.4	5.3459	8.0929	13.91442	1.05877	5.91847	0.07075	11.74933	0.79124	4.253484	0.134984	3.421264	0.237818
-0.2	2.8362	4.2211	5.934662	0.734041	2.560699	0.065268	4.794064	0.463828	1.926938	0.215409	1.705372	0.267899

sag_ratio1												
**Step (nA)**	**Experiment mean**	**Experiment std**	**C10 value**	**C10 error**	**CP15 value**	**CP15 error**	**Tu19 value**	**Tu19 error**	**To21 value**	**To21 error**	**M18 value**	**M18 error**
-1	0.2087	0.0645	0.37262	2.541388	0.411113	3.138189	0.335229	1.961684	0.239728	0.481059	0.223746	0.233264
-0.8	0.2082	0.064	0.406845	3.103836	0.437601	3.584394	0.375204	2.609435	0.253672	0.710507	0.228003	0.309414
-0.6	0.2144	0.0623	0.417225	3.255624	0.42893	3.443502	0.389323	2.807746	0.258923	0.714653	0.227847	0.215841
-0.4	0.2526	0.1541	0.397308	0.939053	0.397963	0.943305	0.371267	0.770067	0.254387	0.011595	0.22527	0.17735
-0.2	0.257	0.0941	0.333225	0.81004	0.385595	1.366581	0.306937	0.53068	0.24011	0.179485	0.225017	0.339878

sag_ratio2												
**Step (nA)**	**Experiment mean**	**Experiment std**	**C10 value**	**C10 error**	**CP15 value**	**CP15 error**	**Tu19 value**	**Tu19 error**	**To21 value**	**To21 error**	**M18 value**	**M18 error**
-1	0.7913	0.0645	0.62738	2.541388	0.588887	3.138189	0.664771	1.961684	0.760272	0.481059	0.776254	0.233264
-0.8	0.7918	0.064	0.593155	3.103836	0.562399	3.584394	0.624796	2.609435	0.746328	0.710507	0.771997	0.309414
-0.6	0.7856	0.0623	0.582775	3.255624	0.57107	3.443502	0.610677	2.807746	0.741077	0.714653	0.772153	0.215841
-0.4	0.7474	0.1541	0.602692	0.939053	0.602037	0.943305	0.628733	0.770067	0.745613	0.011595	0.77473	0.17735
-0.2	0.743	0.0941	0.666775	0.81004	0.614405	1.366581	0.693063	0.53068	0.75989	0.179485	0.774983	0.339878

steady_state_hyper												
**Step (nA)**	**Experiment mean**	**Experiment std**	**C10 value**	**C10 error**	**CP15 value**	**CP15 error**	**Tu19 value**	**Tu19 error**	**To21 value**	**To21 error**	**M18 value**	**M18 error**
-1	-93.2488	7.4089	-113.8	2.773865	-94.7232	0.199005	-114.235	2.83262	-105.733	1.68497	-98.8149	0.751271
-0.8	-89.0217	6.3605	-102.963	2.191916	-87.0245	0.314008	-103.064	2.207658	-98.3877	1.472522	-92.9541	0.618254
-0.6	-84.475	5.6016	-93.5865	1.626593	-80.728	0.668919	-93.5447	1.619118	-91.4692	1.248603	-87.1909	0.484847
-0.4	-79.8991	4.9027	-84.8975	1.019515	-75.2591	0.946413	-84.8709	1.014085	-84.8682	1.013534	-81.3991	0.305945
-0.2	-75.0316	4.5793	-75.6653	0.138378	-70.3858	1.014512	-75.7986	0.167499	-78.4994	0.757272	-75.5064	0.103679

steady_state_voltage												
**Step (nA)**	**Experiment mean**	**Experiment std**	**C10 value**	**C10 error**	**CP15 value**	**CP15 error**	**Tu19 value**	**Tu19 error**	**To21 value**	**To21 error**	**M18 value**	**M18 error**
-1	-69.5976	3.9296	-62.4239	1.825554	-65.6239	1.011228	-63.8388	1.465497	-71.425	0.465036	-68.4643	0.288407
-0.8	-69.6693	3.8566	-62.4762	1.865148	-65.6313	1.047033	-63.7841	1.52602	-71.5044	0.475843	-68.5947	0.278649
-0.6	-69.5911	3.989	-62.6098	1.750147	-65.7119	0.972474	-63.8513	1.438906	-71.6485	0.515776	-68.7731	0.205076
-0.4	-69.5138	4.1133	-62.7529	1.643677	-65.8523	0.890152	-64.1807	1.296552	-71.8579	0.569886	-69.004	0.12393
-0.2	-69.454	4.2827	-63.3324	1.429384	-66.0392	0.797343	-64.6143	1.130061	-72.1189	0.622241	-69.2908	0.038097
0.2	-69.5194	4.2995	-63.1246	1.487343	-66.8658	0.617196	-65.019	1.046737	-72.6744	0.733813	-70.0288	0.118477

0.4	-69.9092	4.3278	-62.4665	1.71975	-67.7537	0.498056	-65.2082	1.086224	-72.9296	0.697906	-70.5483	0.147685
0.6	-70.4804	4.4183	-63.1015	1.670079	-69.4552	0.232045	-65.1616	1.203817	-73.1886	0.612942	-70.9008	0.095145
0.8	-71.0353	4.5287	-63.0051	1.773178	-70.3308	0.155573	-65.0142	1.329549	-73.7491	0.599241	-71.3203	0.062926
1	-71.4634	4.6282	-62.8556	1.859862	-68.8692	0.560524	-64.8954	1.419117	-75.1967	0.806631	-72.2052	0.160283

**steady_state_voltage_stimed**												
**Step (nA)**	**Experiment mean**	**Experiment std**	**C10 value**	**C10 error**	**CP15 value**	**CP15 error**	**Tu19 value**	**Tu19 error**	**To21 value**	**To21 error**	**M18 value**	**M18 error**
-1	-93.3319	7.415	-113.8	2.760376	-94.7232	0.187634	-114.235	2.819082	-105.732	1.672252	-98.8146	0.739403
-0.8	-89.0851	6.3924	-102.963	2.17106	-87.0245	0.322359	-103.064	2.186724	-98.3873	1.455193	-92.954	0.605235
-0.6	-84.4268	5.6363	-93.5865	1.625131	-80.728	0.656249	-93.5447	1.617702	-91.4691	1.24946	-87.191	0.490429
-0.4	-79.8923	4.9452	-84.8975	1.012128	-75.2591	0.936904	-84.8709	1.006745	-84.8683	1.006224	-81.3993	0.304739
-0.2	-75.0121	4.6192	-75.6653	0.141404	-70.3859	1.001517	-75.7986	0.170274	-78.4995	0.754979	-75.5067	0.107067

**voltage_after_stim**												
**Step (nA)**	**Experiment mean**	**Experiment std**	**C10 value**	**C10 error**	**CP15 value**	**CP15 error**	**Tu19 value**	**Tu19 error**	**To21 value**	**To21 error**	**M18 value**	**M18 error**
-1	-69.3894	3.9679	-63.5579	1.469665	-66.1671	0.812099	-64.746	1.170237	-71.9566	0.646996	-69.0578	0.083559
-0.8	-69.466	3.8948	-63.6235	1.50008	-66.1689	0.846545	-64.8247	1.19166	-72.0032	0.651427	-69.1014	0.093622
-0.6	-69.3704	3.9926	-63.7122	1.417173	-66.1715	0.801202	-64.5498	1.207395	-72.0678	0.675604	-69.1682	0.050646
-0.4	-69.2686	4.1337	-63.85	1.31083	-66.1808	0.746991	-64.6739	1.111534	-72.1548	0.698212	-69.2685	1.8E-05
-0.2	-69.2831	4.3611	-63.6983	1.280596	-66.2059	0.705601	-64.8275	1.021664	-72.2665	0.684092	-69.418	0.030922
0.2	-69.6822	4.3321	-63.4454	1.439664	-66.6499	0.69997	-65.2957	1.012562	-72.5532	0.662728	-69.922	0.055357
0.4	-70.1913	4.4134	-63.2654	1.569287	-67.333	0.647648	-65.4486	1.074607	-72.7184	0.572587	-70.3563	0.037392
0.6	-70.7661	4.4566	-63.2193	1.693388	-69.8526	0.204976	-65.4991	1.181848	-72.9096	0.480962	-70.5052	0.058535
0.8	-71.3324	4.5909	-63.1756	1.776735	-70.9258	0.088557	-65.5154	1.267082	-73.1969	0.406123	-70.724	0.132533
1	-71.6974	4.6722	-63.1406	1.831435	-68.4495	0.695164	-65.4901	1.328552	-75.0332	0.71397	-71.147	0.117812

voltage_base													
**Step (nA)**	**Experiment mean**	**Experiment std**	**C10 value**	**C10 error**	**CP15 value**	**CP15 error**	**Tu19 value**	**Tu19 error**	**To21 value**	**To21 error**	**M18 value**	**M18 error**
-1	-69.6021	4.3921	-63.7901	1.323274	-66.3057	0.750531	-64.9736	1.053815	-72.4012	0.63731	-69.6332	0.007079
-0.8	-69.5867	4.3825	-63.7901	1.322659	-66.3057	0.748661	-64.9736	1.052609	-72.4012	0.64222	-69.6332	0.010608
-0.6	-69.4911	4.4034	-63.7901	1.294671	-66.3057	0.723397	-64.9736	1.025903	-72.4012	0.660882	-69.6332	0.032268
-0.4	-69.3713	4.352	-63.7901	1.282434	-66.3057	0.704413	-64.9736	1.010492	-72.4012	0.696215	-69.6332	0.060177
-0.2	-69.3718	4.3803	-63.7901	1.274263	-66.3057	0.699976	-64.9736	1.004077	-72.4012	0.691603	-69.6332	0.059674
0.2	-69.2003	4.4582	-63.7901	1.213528	-66.3057	0.649277	-64.9736	0.948064	-72.4012	0.717986	-69.6332	0.0971
0.4	-69.2273	4.4598	-63.7901	1.219147	-66.3057	0.655098	-64.9736	0.953778	-72.4012	0.711675	-69.6332	0.091011
0.6	-69.5721	4.4253	-63.7901	1.306567	-66.3057	0.738121	-64.9736	1.03913	-72.4012	0.639307	-69.6332	0.013805
0.8	-69.7343	4.4319	-63.7901	1.34122	-66.3057	0.77362	-64.9736	1.07418	-72.4012	0.601757	-69.6332	0.022814
1	-69.931	4.5857	-63.7901	1.339131	-66.3057	0.790568	-64.9736	1.081048	-72.4012	0.53868	-69.6332	0.064943

voltage_deflection													
**Step (nA)**	**Experiment mean**	**Experiment std**	**C10 value**	**C10 error**	**CP15 value**	**CP15 error**	**Tu19 value**	**Tu19 error**	**To21 value**	**To21 error**	**M18 value**	**M18 error**
-1	-23.6514	5.7217	-50.0036	4.605667	-28.4319	0.835499	-49.2638	4.476368	-33.1899	1.667067	-29.0974	0.95181
-0.8	-19.3666	4.7267	-39.1669	4.189041	-20.7331	0.289107	-38.092	3.961612	-25.8449	1.370582	-23.2366	0.818747
-0.6	-15.0155	3.5413	-29.7901	4.172078	-14.4366	0.163457	-28.5731	3.828423	-18.9264	1.104371	-17.4734	0.694061
-0.4	-10.4888	2.5469	-21.101	4.166724	-8.96779	0.5972	-19.8993	3.694883	-12.3254	0.721103	-11.6815	0.468298
-0.2	-5.6461	1.5728	-11.8688	3.956466	-4.09451	0.986514	-10.8271	3.294106	-5.9566	0.197419	-5.78882	0.090745

voltage_deflection_begin													
**Step (nA)**	**Experiment mean**	**Experiment std**	**C10 value**	**C10 error**	**CP15 value**	**CP15 error**	**Tu19 value**	**Tu19 error**	**To21 value**	**To21 error**	**M18 value**	**M18 error**
-1	-27.9145	7.2572	-55.525	3.804569	-37.4865	1.318972	-57.2856	4.047173	-35.5329	1.049777	-32.1209	0.579624
-0.8	-22.9462	6.025	-44.3134	3.546416	-27.8917	0.820833	-45.6701	3.771608	-28.1468	0.863171	-25.8871	0.488108

-0.6	-17.5106	4.5662	-33.9076	3.590952	-18.5854	0.235374	-34.6453	3.752512	-20.8893	0.739934	-19.6405	0.466457
-0.4	-11.9487	3.1191	-23.9453	3.846161	-10.6276	0.423554	-24.0022	3.864406	-13.7577	0.579982	-13.2831	0.427818
-0.2	-6.1813	1.6983	-13.6942	4.423765	-4.54291	0.964725	-13.0238	4.029024	-6.71017	0.311411	-6.69751	0.30396

**voltage_deflection_vb_ssse**												
**Step (nA)**	**Experiment mean**	**Experiment std**	**C10 value**	**C10 error**	**CP15 value**	**CP15 error**	**Tu19 value**	**Tu19 error**	**To21 value**	**To21 error**	**M18 value**	**M18 error**
-1	-23.7298	5.8063	-50.0099	4.526143	-28.4175	0.80735	-49.2618	4.397285	-33.3304	1.653484	-29.1814	0.938908
-0.8	-19.4985	4.6597	-39.1732	4.222318	-20.7188	0.261875	-38.0899	3.989822	-25.986	1.392267	-23.3208	0.820292
-0.6	-14.9357	3.6064	-29.7964	4.12064	-14.4223	0.142361	-28.571	3.780865	-19.0679	1.145797	-17.5578	0.727073
-0.4	-10.521	2.5677	-21.1073	4.122883	-8.95343	0.610496	-19.8972	3.651601	-12.4671	0.757898	-11.7661	0.48491
-0.2	-5.6403	1.549	-11.8751	4.025065	-4.0802	1.007167	-10.825	3.34712	-6.09827	0.295658	-5.87347	0.15053

**Spikecount**												
**Step (nA)**	**Experiment mean**	**Experiment std**	**C10 value**	**C10 error**	**CP15 value**	**CP15 error**	**Tu19 value**	**Tu19 error**	**To21 value**	**To21 error**	**M18 value**	**M18 error**
0.2	1.4774	2.0734	32	14.72104	0	0.712549	23	10.38034	0	0.712549	0	0.712549
0.4	3.9763	3.3616	47	12.79858	1	0.885382	37	9.823804	0	1.182859	3	0.290427
0.6	6.6785	4.0206	56	12.2672	1	1.412351	45	9.531289	1	1.412351	9	0.577401
0.8	9.3075	4.1665	62	12.64671	1	1.99388	54	10.72663	4	1.273851	12	0.646226
1	11.325	4.6197	73	13.35043	3	1.802065	59	10.31993	15	0.795506	16	1.01197

**Spikecount_stimint**												
**Step (nA)**	**Experiment mean**	**Experiment std**	**C10 value**	**C10 error**	**CP15 value**	**CP15 error**	**Tu19 value**	**Tu19 error**	**To21 value**	**To21 error**	**M18 value**	**M18 error**
0.2	1.4774	2.0734	32	14.72104	0	0.712549	23	10.38034	0	0.712549	0	0.712549
0.4	3.9763	3.3616	46	12.5011	1	0.885382	37	9.823804	0	1.182859	3	0.290427
0.6	6.6785	4.0206	56	12.2672	1	1.412351	45	9.531289	1	1.412351	9	0.577401

0.8	9.3075	4.1665	62	12.64671	1	1.99388	54	10.72663	4	1.273851	12	0.646226
1	11.325	4.6197	73	13.35043	3	1.802065	59	10.31993	15	0.795506	16	1.01197

**inv_time_to_first_spike**												
**Step (nA)**	**Experiment mean**	**Experiment std**	**C10 value**	**C10 error**	**CP15 value**	**CP15 error**	**Tu19 value**	**Tu19 error**	**To21 value**	**To21 error**	**M18 value**	**M18 error**
0.2	23.5725	33.9424	217.3913	5.710227	0	0.694485	128.2051	3.082653	0	0.694485	0	0.694485
0.4	59.1824	66.4808	400	5.126557	123.4568	0.966811	285.7143	3.407478	0	0.890218	93.45794	0.515571
0.6	97.5343	89.619	555.5556	5.110761	263.1579	1.848086	400	3.375018	113.6364	0.179672	169.4915	0.802924
0.8	148.3142	114.7306	714.2857	4.933048	370.3704	1.935457	526.3158	3.294689	200	0.450497	250	0.886301
1	196.2319	136.6747	833.3333	4.661444	454.5455	1.889988	625	3.137143	277.7778	0.596642	322.5806	0.924449

**inv_first_ISI**												
**Step (nA)**	**Experiment mean**	**Experiment std**	**C10 value**	**C10 error**	**CP15 value**	**CP15 error**	**Tu19 value**	**Tu19 error**	**To21 value**	**To21 error**	**M18 value**	**M18 error**
0.2	14.0118	23.7191	87.7193	3.107517	0	0.590739	64.51613	2.129268	0	0.590739	0	0.590739
0.4	40.8817	40.4993	131.5789	2.239477	0	1.009442	100	1.459736	0	1.009442	27.70083	0.325459
0.6	68.5665	47.4419	113.6364	0.950001	0	1.445273	128.2051	1.257088	0	1.445273	53.47594	0.318085
0.8	93.8851	46.4426	46.08295	1.029274	0	2.02153	151.5152	1.240888	47.61905	0.996199	72.46377	0.461243
1	116.3015	43.2083	68.96552	1.09553	7.782101	2.511541	185.1852	1.594223	87.7193	0.661498	90.90909	0.587674

**inv_second_ISI**												
**Step (nA)**	**Experiment mean**	**Experiment std**	**C10 value**	**C10 error**	**CP15 value**	**CP15 error**	**Tu19 value**	**Tu19 error**	**To21 value**	**To21 error**	**M18 value**	**M18 error**
0.2	4.5983	8.9653	83.33333	8.782197	0	0.5129	58.47953	6.009975	0	0.5129	0	0.5129
0.4	20.0842	21.6747	212.766	8.889708	0	0.92662	96.15385	3.509605	0	0.92662	7.401925	0.585119
0.6	41.8257	29.8653	238.0952	6.571825	0	1.400478	204.0816	5.432925	0	1.400478	34.60208	0.241873
0.8	62.9663	30.2882	178.5714	3.816837	0	2.078905	222.2222	5.258019	6.23053	1.873197	54.94505	0.264831

1	80.6491	31.1475	250	5.437062	5.574136	2.410305	112.3596	1.018074	82.64463	0.064067	74.07407	0.211093

inv_third_ISI												
**Step (nA)**	**Experiment mean**	**Experiment std**	**C10 value**	**C10 error**	**CP15 value**	**CP15 error**	**Tu19 value**	**Tu19 error**	**To21 value**	**To21 error**	**M18 value**	**M18 error**
0.2	2.4873	5.8108	78.74016	13.12261	0	0.428048	56.49718	9.29474	0	0.428048	0	0.428048
0.4	12.5847	14.2164	232.5581	15.47322	0	0.885224	92.59259	5.627859	0	0.885224	0	0.885224
0.6	25.5306	19.4195	238.0952	10.94594	0	1.314689	117.6471	4.743503	0	1.314689	21.36752	0.214376
0.8	39.8159	20.768	144.9275	5.061231	0	1.917175	196.0784	7.524197	8.673027	1.499561	38.16794	0.079351
1	53.2365	20.1677	227.2727	8.629453	0	2.639691	106.383	2.635228	64.10256	0.538785	56.81818	0.177595

inv_fourth_ISI												
**Step (nA)**	**Experiment mean**	**Experiment std**	**C10 value**	**C10 error**	**CP15 value**	**CP15 error**	**Tu19 value**	**Tu19 error**	**To21 value**	**To21 error**	**M18 value**	**M18 error**
0.2	1.2653	4.6328	77.51938	16.45961	0	0.273118	56.17978	11.85341	0	0.273118	0	0.273118
0.4	8.7874	12.1634	227.2727	17.96252	0	0.722446	91.74312	6.820109	0	0.722446	0	0.722446
0.6	18.5295	16.8817	181.8182	9.672526	0	1.097609	57.14286	2.287291	0	1.097609	19.53125	0.059339
0.8	30.6315	16.9188	142.8571	6.633192	0	1.810501	250	12.96596	0	1.810501	29.15452	0.087298
1	40.7579	15.8266	200	10.06167	0	2.575278	172.4138	8.318647	48.30918	0.477126	46.2963	0.349942

inv_fifth_ISI												
**Step (nA)**	**Experiment mean**	**Experiment std**	**C10 value**	**C10 error**	**CP15 value**	**CP15 error**	**Tu19 value**	**Tu19 error**	**To21 value**	**To21 error**	**M18 value**	**M18 error**
0.2	0.6903	3.5827	77.51938	21.44446	0	0.192676	56.17978	15.48817	0	0.192676	0	0.192676
0.4	6.3819	10.3146	151.5152	14.07066	0	0.618725	90.90909	8.194907	0	0.618725	0	0.618725
0.6	14.6701	15.6837	138.8889	7.920248	0	0.935372	99.0099	5.377545	0	0.935372	19.49318	0.307522
0.8	24.9452	18.1057	147.0588	6.744485	0	1.377754	86.95652	3.424961	0	1.377754	26.38522	0.079534
1	34.2529	17.3915	166.6667	7.613706	0	1.96952	138.8889	6.016502	38.75969	0.259138	39.84064	0.321291

inv_last_ISI													
**Step (nA)**	**Experiment mean**	**Experiment std**	**C10 value**	**C10 error**	**CP15 value**	**CP15 error**	**Tu19 value**	**Tu19 error**	**To21 value**	**To21 error**	**M18 value**	**M18 error**
0.2	6.2691	17.9501	78.125	4.003092	0	0.349252	55.55556	2.745748	0	0.349252	0	0.349252
0.4	8.1811	6.6758	111.1111	15.41838	0	1.225486	90.90909	12.39222	0	1.225486	7.401925	0.116716
0.6	12.8949	7.5643	138.8889	16.6564	0	1.704705	113.6364	13.31802	0	1.704705	19.49318	0.872292
0.8	17.3649	6.667	163.9344	21.98433	0	2.604605	133.3333	17.3944	8.673027	1.303716	25.97403	1.291304
1	21.0827	7.7745	185.1852	21.10779	5.574136	1.994799	149.2537	16.48608	30.95975	1.270442	33.44482	1.590085

maximum_voltage												
**Step (nA)**	**Experiment mean**	**Experiment std**	**C10 value**	**C10 error**	**CP15 value**	**CP15 error**	**Tu19 value**	**Tu19 error**	**To21 value**	**To21 error**	**M18 value**	**M18 error**
0.2	-19.8966	44.7483	17.05651	0.825799	-60.6518	0.910765	14.41184	0.766698	-65.2766	1.014116	-61.7413	0.935113
0.4	9.3048	37.7778	17.13169	0.207182	11.18398	0.049743	15.16358	0.155085	-58.5645	1.79654	38.04696	0.760821
0.6	29.5141	10.1374	17.06687	1.227852	10.89965	1.836216	14.74341	1.457049	25.16374	0.42914	38.10062	0.847014
0.8	30.4901	7.6295	17.80262	1.66295	11.63042	2.471941	15.49157	1.96586	25.32588	0.676875	37.44165	0.911141
1	29.4959	8.0014	19.21274	1.28517	12.3398	2.144138	16.27486	1.652341	23.18031	0.78931	37.24258	0.968165

maximum_voltage_from_voltagebase												
**Step (nA)**	**Experiment mean**	**Experiment std**	**C10 value**	**C10 error**	**CP15 value**	**CP15 error**	**Tu19 value**	**Tu19 error**	**To21 value**	**To21 error**	**M18 value**	**M18 error**
0.2	49.3037	44.0619	80.84666	0.715878	5.653893	0.990647	79.38548	0.682716	7.124662	0.957268	7.891874	0.939856
0.4	78.5321	37.0959	80.92184	0.06442	77.48968	0.028101	80.13722	0.043269	13.83669	1.744004	107.6802	0.785749
0.6	99.0862	11.1674	80.85702	1.632357	77.20534	1.959351	79.71705	1.734437	97.56497	0.136221	107.7338	0.774362
0.8	100.2244	7.9104	81.59277	2.355334	77.93612	2.817592	80.46521	2.497875	97.72711	0.315697	107.0748	0.866004
1	99.4269	7.9299	83.00289	2.07115	78.64549	2.62064	81.2485	2.292387	95.58154	0.484919	106.8758	0.939339

number_initial_spikes													
**Step (nA)**	**Experiment mean**	**Experiment std**	**C10 value**	**C10 error**	**CP15 value**	**CP15 error**	**Tu19 value**	**Tu19 error**	**To21 value**	**To21 error**	**M18 value**	**M18 error**
0.2	0.4903	0.7617	3	3.294867	0	0.643692	2	1.982014	0	0.643692	0	0.643692
0.4	1.0097	1.0759	6	4.638256	1	0.009016	4	2.779348	0	0.93847	1	0.009016
0.6	1.7879	1.2638	6	3.332885	1	0.623437	4	1.750356	1	0.623437	2	0.167827
0.8	2.35	1.2058	4	1.368386	1	1.119589	6	3.027036	2	0.290264	3	0.539061
1	3.0742	0.9131	6	3.204249	1	2.271602	6	3.204249	3	0.081262	3	0.081262

AHP1_depth_from_peak												
**Step (nA)**	**Experiment mean**	**Experiment std**	**C10 value**	**C10 error**	**CP15 value**	**CP15 error**	**Tu19 value**	**Tu19 error**	**To21 value**	**To21 error**	**M18 value**	**M18 error**
0.8	92.1793	4.917	53.45427	7.875744	79.92442	2.49235	60.7462	6.39274	80.74757	2.324939	95.95562	0.768012
1	91.6409	4.9559	52.86194	7.824808	78.80065	2.590902	55.6124	7.26982	72.99292	3.762783	91.81418	0.034965

AHP2_depth_from_peak												
**Step (nA)**	**Experiment mean**	**Experiment std**	**C10 value**	**C10 error**	**CP15 value**	**CP15 error**	**Tu19 value**	**Tu19 error**	**To21 value**	**To21 error**	**M18 value**	**M18 error**
0.8	82.8879	8.2237	42.47975	4.913621			47.16344	4.344086	78.57216	0.524792	98.16248	1.857386
1	79.8599	8.0481	18.95082	7.568132	77.40772	0.304691	23.71602	6.976042	60.52692	2.402179	90.82322	1.362225

AHP_depth												
**Step (nA)**	**Experiment mean**	**Experiment std**	**C10 value**	**C10 error**	**CP15 value**	**CP15 error**	**Tu19 value**	**Tu19 error**	**To21 value**	**To21 error**	**M18 value**	**M18 error**
0.8	11.673	4.0636	15.32397	0.898458	-1.9883	3.361871	14.67699	0.739244	14.94095	0.804202	5.458804	1.529234
1	12.5085	4.6265	18.29296	1.250289	-0.92749	2.904137	16.84044	0.936331	17.17374	1.008374	7.58971	1.063177

AHP_depth_abs

Step (nA)	Experiment mean	Experiment std	C10 value	C10 error	CP15 value	CP15 error	Tu19 value	Tu19 error	To21 value	To21 error	M18 value	M18 error
0.8	-58.0613	5.8413	-48.4662	1.642635	-68.294	1.751783	-50.2966	1.329268	-57.4603	0.102893	-64.1744	1.046529
1	-57.4225	6.6545	-45.4972	1.792068	-67.2332	1.474293	-48.1332	1.395942	-55.2275	0.329854	-62.0435	0.694414

AHP_depth_diff												
Step (nA)	Experiment mean	Experiment std	C10 value	C10 error	CP15 value	CP15 error	Tu19 value	Tu19 error	To21 value	To21 error	M18 value	M18 error
0.8	0.4578	0.2903	-0.20813	2.293947			-0.40245	2.963298	-1.07706	5.287155	-0.59906	3.640572
1	0.4563	0.3038	-0.45688	3.005843	-0.61474	3.525482	-0.15819	2.022668	-0.48096	3.085114	-0.58831	3.438495

AHP_depth_from_peak												
Step (nA)	Experiment mean	Experiment std	C10 value	C10 error	CP15 value	CP15 error	Tu19 value	Tu19 error	To21 value	To21 error	M18 value	M18 error
0.8	81.4373	8.7435	62.94088	2.115448	79.92442	0.173029	62.85901	2.124811	79.62343	0.207453	101.2524	2.266267
1	77.2394	9.281	54.84437	2.412998	77.77065	0.057241	60.23919	1.831722	74.95904	0.245702	98.49897	2.290655

AHP_time_from_peak												
Step (nA)	Experiment mean	Experiment std	C10 value	C10 error	CP15 value	CP15 error	Tu19 value	Tu19 error	To21 value	To21 error	M18 value	M18 error
0.8	2.1146	0.2597	4.191935	7.998981	8.5	24.5876	4.575926	9.477574	7.725	21.60339	6.908333	18.45873
1	2.2066	0.2753	3.846575	5.957048	7.3	18.50127	4.354237	7.80108	7.066667	17.65371	6.5375	15.73157

AP1_peak												
Step (nA)	Experiment mean	Experiment std	C10 value	C10 error	CP15 value	CP15 error	Tu19 value	Tu19 error	To21 value	To21 error	M18 value	M18 error
0.8	30.4901	7.6295	17.80262	1.66295	11.63042	2.471941	15.49157	1.96586	25.32588	0.676875	37.26235	0.887641
1	29.4878	8.0018	19.21274	1.284094	12.3398	2.143018	16.27486	1.651246	23.18031	0.788258	37.24258	0.969129

AP2_AP1_peak_diff													
**Step (nA)**	**Experiment mean**	**Experiment std**	**C10 value**	**C10 error**	**CP15 value**	**CP15 error**	**Tu19 value**	**Tu19 error**	**To21 value**	**To21 error**	**M18 value**	**M18 error**
0.8	-4.2233	2.8446	-24.0869	6.982932			-6.7832	0.899915	-4.02435	0.069939	-0.32377	1.370853
1	-5.6801	3.1828	-38.2749	10.24093	-2.48044	1.005297	-27.2139	6.765666	-13.5833	2.483103	-3.51317	0.680826

AP2_amp												
**Step (nA)**	**Experiment mean**	**Experiment std**	**C10 value**	**C10 error**	**CP15 value**	**CP15 error**	**Tu19 value**	**Tu19 error**	**To21 value**	**To21 error**	**M18 value**	**M18 error**
0.8	77.7102	7.0102	24.59139	7.57736			51.95838	3.673479	67.87002	1.403694	83.10238	0.76919
1	75.764	7.0631	11.37635	9.116061	64.26596	1.627903	27.70747	6.803887	53.06723	3.213429	78.59207	0.4004

AP2_begin_voltage												
**Step (nA)**	**Experiment mean**	**Experiment std**	**C10 value**	**C10 error**	**CP15 value**	**CP15 error**	**Tu19 value**	**Tu19 error**	**To21 value**	**To21 error**	**M18 value**	**M18 error**
0.8	-51.4433	7.0197	-30.8757	2.92998			-43.25	1.167186	-46.5685	0.694447	-46.1638	0.752099
1	-51.9563	7.6988	-30.4386	2.794949	-54.4066	0.318271	-38.6465	1.72882	-43.4702	1.102258	-44.8627	0.921396

AP2_begin_width												
**Step (nA)**	**Experiment mean**	**Experiment std**	**C10 value**	**C10 error**	**CP15 value**	**CP15 error**	**Tu19 value**	**Tu19 error**	**To21 value**	**To21 error**	**M18 value**	**M18 error**
0.8	2.0749	0.537	1.7	0.698138			4.2	3.957356	2.5	0.79162	3.1	1.908939
1	2.2177	0.5446	2.1	0.216122	3.3	1.98733	3.3	1.98733	2.1	0.216122	3.2	1.803709

AP2_peak												
**Step (nA)**	**Experiment mean**	**Experiment std**	**C10 value**	**C10 error**	**CP15 value**	**CP15 error**	**Tu19 value**	**Tu19 error**	**To21 value**	**To21 error**	**M18 value**	**M18 error**

0.8	26.2669	9.5008	-6.28433	3.426157			8.708374	1.84811	21.30153	0.522627	36.93858	1.123241
1	23.8077	10.123	-19.0622	4.234901	9.859355	1.377886	-10.939	3.432451	9.596994	1.403804	33.72941	0.980115

**AP2_width**												
**Step (nA)**	**Experiment mean**	**Experiment std**	**C10 value**	**C10 error**	**CP15 value**	**CP15 error**	**Tu19 value**	**Tu19 error**	**To21 value**	**To21 error**	**M18 value**	**M18 error**
0.8	0.7907	0.1227	18.333	142.969			1.233277	3.606982	0.6627	1.043198	1.23267	3.602039
1	0.8552	0.1207	10.95451	83.67283	1.540434	5.677166	1.18124	2.70124	0.758359	0.802326	1.242332	3.207392

**AP_amplitude**												
**Step (nA)**	**Experiment mean**	**Experiment std**	**C10 value**	**C10 error**	**CP15 value**	**CP15 error**	**Tu19 value**	**Tu19 error**	**To21 value**	**To21 error**	**M18 value**	**M18 error**
0.8	72.712	8.9022	58.66097	1.578378			57.24089	1.737898	68.10506	0.517506	83.58688	1.221594
1	68.9557	9.4164	51.47802	1.856089	64.17996	0.507173	55.67496	1.410384	64.58947	0.463683	82.1769	1.404061

**AP_amplitude_change**												
**Step (nA)**	**Experiment mean**	**Experiment std**	**C10 value**	**C10 error**	**CP15 value**	**CP15 error**	**Tu19 value**	**Tu19 error**	**To21 value**	**To21 error**	**M18 value**	**M18 error**
0.8	-0.1681	0.0809	-0.28105	1.39619			-0.28863	1.489811	-0.30311	1.668848	-0.21805	0.61744
1	-0.2138	0.0925	-0.3798	1.794642	-0.18393	0.322882	-0.31476	1.091428	-0.32425	1.194026	-0.2311	0.187015

**AP_amplitude_from_voltagebase**												
**Step (nA)**	**Experiment mean**	**Experiment std**	**C10 value**	**C10 error**	**CP15 value**	**CP15 error**	**Tu19 value**	**Tu19 error**	**To21 value**	**To21 error**	**M18 value**	**M18 error**
0.8	93.084	11.1059	78.26485	1.334349	77.93612	1.363949	77.536	1.399976	94.56439	0.133297	106.7112	1.227024
1	89.7448	11.789	73.13733	1.408726	76.84316	1.094379	77.07963	1.074321	92.13278	0.20256	106.0887	1.386367

**AP_begin_voltage**

Step (nA)	Experiment mean	Experiment std	C10 value	C10 error	CP15 value	CP15 error	Tu19 value	Tu19 error	To21 value	To21 error	M18 value	M18 error
0.8	-49.3576	6.4407	-44.2408	0.794445			-44.7338	0.717905	-46.9961	0.366646	-46.5256	0.439701
1	-49.1419	7.24	-42.2679	0.949453	-54.5437	0.746098	-43.6409	0.759813	-45.1043	0.557686	-45.7739	0.465196

AP_begin_width												
Step (nA)	Experiment mean	Experiment std	C10 value	C10 error	CP15 value	CP15 error	Tu19 value	Tu19 error	To21 value	To21 error	M18 value	M18 error
0.8	1.7972	0.3422	3.972131	6.355731			4.028302	6.519877	2.433333	1.858952	3.027273	3.594602
1	1.8931	0.3592	3.859722	5.475006	3.3	3.916759	4.07931	6.086332	2.364286	1.311764	3.033333	3.174369

AP_duration												
Step (nA)	Experiment mean	Experiment std	C10 value	C10 error	CP15 value	CP15 error	Tu19 value	Tu19 error	To21 value	To21 error	M18 value	M18 error
0.8	1.9937	0.1717	2.732787	4.304525			2.924528	5.421248	2.1	0.619103	3.163636	6.81384
1	2.0392	0.1693	2.198611	0.94159	3.55	8.923804	2.886207	5.002994	2.1	0.359126	3.153333	6.580823

AP_duration_change												
Step (nA)	Experiment mean	Experiment std	C10 value	C10 error	CP15 value	CP15 error	Tu19 value	Tu19 error	To21 value	To21 error	M18 value	M18 error
0.8	0.1391	0.0966	-0.34518	5.013203			-0.28565	4.39697	-0.68182	8.498118	-0.49524	6.566647
1	0.1828	0.0904	-0.40362	6.486966	-0.33333	5.70944	-0.21053	4.350955	-0.57846	8.421035	-0.39148	6.352694

AP_duration_half_width												
Step (nA)	Experiment mean	Experiment std	C10 value	C10 error	CP15 value	CP15 error	Tu19 value	Tu19 error	To21 value	To21 error	M18 value	M18 error
0.8	0.8029	0.0943	1.262295	4.871634			1.469811	7.07223	0.7	1.091198	1.236364	4.596645
1	0.8315	0.0942	1.211111	4.029842	1.4	6.035032	1.598276	8.139871	0.7	1.395966	1.253333	4.478061

AP_duration_half_width_change												
**Step (nA)**	**Experiment mean**	**Experiment std**	**C10 value**	**C10 error**	**CP15 value**	**CP15 error**	**Tu19 value**	**Tu19 error**	**To21 value**	**To21 error**	**M18 value**	**M18 error**
0.8	0.1537	0.1399	-0.46875	4.449249			-0.41308	4.0513	-0.3	3.243031	-0.12143	1.966609
1	0.2238	0.1138	-0.57638	7.031471	-0.22222	3.919352	-0.35298	5.068387	-0.3	4.602812	-0.16667	3.431166

AP_fall_rate												
**Step (nA)**	**Experiment mean**	**Experiment std**	**C10 value**	**C10 error**	**CP15 value**	**CP15 error**	**Tu19 value**	**Tu19 error**	**To21 value**	**To21 error**	**M18 value**	**M18 error**
0.8	-52.9764	9.141	-15.8216	4.064629	-22.4207	3.342705	-20.1243	3.593926	-43.7642	1.007789	-35.2976	1.93401
1	-48.6185	9.8823	-13.0491	3.599303	-23.9712	2.494082	-19.7777	2.918431	-43.3879	0.529289	-35.11	1.366939

AP_fall_time												
**Step (nA)**	**Experiment mean**	**Experiment std**	**C10 value**	**C10 error**	**CP15 value**	**CP15 error**	**Tu19 value**	**Tu19 error**	**To21 value**	**To21 error**	**M18 value**	**M18 error**
0.8	1.5073	0.1191	1.708065	1.68568	3.1	13.3728	2.074074	4.758808	1.5	0.061293	2.433333	7.775259
1	1.5263	0.142	1.265753	1.834835	2.9	9.673944	1.967797	3.109131	1.44	0.607746	2.4	6.152817

AP_height												
**Step (nA)**	**Experiment mean**	**Experiment std**	**C10 value**	**C10 error**	**CP15 value**	**CP15 error**	**Tu19 value**	**Tu19 error**	**To21 value**	**To21 error**	**M18 value**	**M18 error**
0.8	23.3497	10.9233	14.4747	0.812483	11.63042	1.072869	12.56237	0.987553	22.16316	0.108625	37.07802	1.256792
1	19.8138	11.8553	9.347181	0.882864	10.53747	0.782463	12.10599	0.650158	19.73156	0.006937	36.45549	1.403734

AP_rise_rate												
**Step (nA)**	**Experiment mean**	**Experiment std**	**C10 value**	**C10 error**	**CP15 value**	**CP15 error**	**Tu19 value**	**Tu19 error**	**To21 value**	**To21 error**	**M18 value**	**M18 error**

0.8	160.9801	45.3824	76.48788	1.861784			79.12712	1.803628	108.1219	1.164729	116.6858	0.976025
1	146.3948	43.2626	62.81344	1.931954	80.22495	1.529493	74.09294	1.671232	99.49018	1.084184	112.5158	0.783101

**AP_rise_rate_change**												
**Step (nA)**	**Experiment mean**	**Experiment std**	**C10 value**	**C10 error**	**CP15 value**	**CP15 error**	**Tu19 value**	**Tu19 error**	**To21 value**	**To21 error**	**M18 value**	**M18 error**
0.8	-0.2631	0.1487	0.333875	4.01463			0.870629	7.624274	4.817439	34.16637	3.358744	24.35672
1	-0.3416	0.1404	-0.07933	1.868044	1.241176	11.27333	0.47272	5.800002	2.843277	22.68431	2.293171	18.76617

**AP_rise_time**												
**Step (nA)**	**Experiment mean**	**Experiment std**	**C10 value**	**C10 error**	**CP15 value**	**CP15 error**	**Tu19 value**	**Tu19 error**	**To21 value**	**To21 error**	**M18 value**	**M18 error**
0.8	0.4864	0.0935	1.036066	5.878776			0.85283	3.919039	0.633333	1.57148	0.718182	2.47895
1	0.5129	0.1001	0.947222	4.338883	0.8	2.868132	0.92069	4.073823	0.657143	1.440988	0.733333	2.202131

**AP_width**												
**Step (nA)**	**Experiment mean**	**Experiment std**	**C10 value**	**C10 error**	**CP15 value**	**CP15 error**	**Tu19 value**	**Tu19 error**	**To21 value**	**To21 error**	**M18 value**	**M18 error**
0.8	0.9107	0.1229	1.883607	7.916245			1.745283	6.790749	0.866667	0.358286	1.572727	5.386715
1	0.9291	0.1458	1.798611	5.963725	1.25	2.20096	1.765517	5.736744	0.821429	0.738487	1.613333	4.692958

**APlast_amp**												
**Step (nA)**	**Experiment mean**	**Experiment std**	**C10 value**	**C10 error**	**CP15 value**	**CP15 error**	**Tu19 value**	**Tu19 error**	**To21 value**	**To21 error**	**M18 value**	**M18 error**
0.8	65.4508	11.0654	58.42831	0.634635	63.44244	0.181499	59.88565	0.502932	68.13201	0.242306	84.06944	1.6826
1	60.9569	12.1796	52.35967	0.705871	64.09396	0.257566	57.85012	0.255081	66.34042	0.442011	82.43395	1.763362

**ISI_values**

Step (nA)	Experiment mean	Experiment std	C10 value	C10 error	CP15 value	CP15 error	Tu19 value	Tu19 error	To21 value	To21 error	M18 value	M18 error
0.8	48.4821	30.9888	6.206667	1.364217			7.496154	1.322605	137.9	2.885491	34.72	0.444099
1	34.5385	12.0706	5.395775	2.414356	179.4	12.00118	6.798246	2.298167	27.68462	0.567816	26.65	0.65353

amp_drop_first_last												
Step (nA)	Experiment mean	Experiment std	C10 value	C10 error	CP15 value	CP15 error	Tu19 value	Tu19 error	To21 value	To21 error	M18 value	M18 error
0.8	11.8027	6.7405	3.260094	1.267355			1.081422	1.590576	4.457614	1.089694	0.353906	1.698508
1	14.9766	7.6991	9.624035	0.69522	2.926542	1.565126	2.886807	1.570287	2.444336	1.627757	0.792784	1.842269

amp_drop_first_second												
Step (nA)	Experiment mean	Experiment std	C10 value	C10 error	CP15 value	CP15 error	Tu19 value	Tu19 error	To21 value	To21 error	M18 value	M18 error
0.8	4.2233	2.8446	24.08695	6.982932			6.783199	0.899915	4.024351	0.069939	0.323771	1.370853
1	5.6801	3.1828	38.27494	10.24093	2.48044	1.005297	27.21386	6.765666	13.58332	2.483103	3.513167	0.680826

amp_drop_second_last												
Step (nA)	Experiment mean	Experiment std	C10 value	C10 error	CP15 value	CP15 error	Tu19 value	Tu19 error	To21 value	To21 error	M18 value	M18 error
0.8	7.5794	5.5013	-20.8269	5.163553			-5.70178	2.414189	0.433264	1.29899	0.030135	1.372269
1	9.2965	6.0702	-28.6509	6.251426	0.446101	1.458008	-24.3271	5.539118	-11.139	3.366526	-2.72038	1.979652

doublet_ISI												
Step (nA)	Experiment mean	Experiment std	C10 value	C10 error	CP15 value	CP15 error	Tu19 value	Tu19 error	To21 value	To21 error	M18 value	M18 error
0.8	15.0424	9.8883	21.7	0.673281			6.6	0.853777	21	0.60249	13.8	0.125643
1	10.1299	4.2819	14.5	1.020598	128.5	27.64429	5.4	1.104626	11.4	0.296621	11	0.203204

fast_AHP													
**Step (nA)**	**Experiment mean**	**Experiment std**	**C10 value**	**C10 error**	**CP15 value**	**CP15 error**	**Tu19 value**	**Tu19 error**	**To21 value**	**To21 error**	**M18 value**	**M18 error**
0.8	8.2685	2.869	4.434988	1.336184			5.360864	1.013467	11.02092	0.959367	18.14957	3.44408
1	7.9004	2.4961	3.106849	1.920416	13.14176	2.09982	4.654415	1.300423	10.47681	1.032174	16.72169	3.534029

fast_AHP_change												
**Step (nA)**	**Experiment mean**	**Experiment std**	**C10 value**	**C10 error**	**CP15 value**	**CP15 error**	**Tu19 value**	**Tu19 error**	**To21 value**	**To21 error**	**M18 value**	**M18 error**
0.8	0.2827	0.8433	-1.14951	1.698339			-1.28196	1.855403	-1.66785	2.312991	-2.6904	3.525552
1												

irregularity_index												
**Step (nA)**	**Experiment mean**	**Experiment std**	**C10 value**	**C10 error**	**CP15 value**	**CP15 error**	**Tu19 value**	**Tu19 error**	**To21 value**	**To21 error**	**M18 value**	**M18 error**
0.8	10.1155	9.5916	0.105	1.043674			0.303846	1.022942	22.6	1.301608	2.05	0.840892
1	7.3445	9.1538	0.08169	0.79342	0	0.802344	0.14386	0.786629	1.569231	0.630915	1.171429	0.674373

max_amp_difference												
**Step (nA)**	**Experiment mean**	**Experiment std**	**C10 value**	**C10 error**	**CP15 value**	**CP15 error**	**Tu19 value**	**Tu19 error**	**To21 value**	**To21 error**	**M18 value**	**M18 error**
0.8	4.8977	2.7676	24.08695	6.933534			27.22874	8.068738	4.024351	0.315562	0.573725	1.562356
1	6.0333	2.8755	38.27494	11.21253	2.48044	1.235562	27.21386	7.36587	13.58332	2.625637	3.513167	0.876416

mean_frequency												
**Step (nA)**	**Experiment mean**	**Experiment std**	**C10 value**	**C10 error**	**CP15 value**	**CP15 error**	**Tu19 value**	**Tu19 error**	**To21 value**	**To21 error**	**M18 value**	**M18 error**

0.8	31.5698	10.6864	156.7636	11.71524	370.3704	31.7039	135.5762	9.732595	13.25381	1.713953	32.87671	0.122297
1	37.5047	9.4431	183.0491	15.41278	9.674299	2.947168	149.5564	11.86599	40.01067	0.265376	41.32231	0.404276

**min_AHP_values**												
**Step (nA)**	**Experiment mean**	**Experiment std**	**C10 value**	**C10 error**	**CP15 value**	**CP15 error**	**Tu19 value**	**Tu19 error**	**To21 value**	**To21 error**	**M18 value**	**M18 error**
0.8	-58.0613	5.8413	-48.4662	1.642635	-68.294	1.751783	-50.2966	1.329268	-57.4603	0.102893	-64.1744	1.046529
1	-57.4225	6.6545	-45.4972	1.792068	-67.2332	1.474293	-48.1332	1.395942	-55.2275	0.329854	-62.0435	0.694414

**min_voltage_between_spikes**												
**Step (nA)**	**Experiment mean**	**Experiment std**	**C10 value**	**C10 error**	**CP15 value**	**CP15 error**	**Tu19 value**	**Tu19 error**	**To21 value**	**To21 error**	**M18 value**	**M18 error**
0.8	-58.9266	5.8058	-48.5823	1.781712			-50.2836	1.488678	-57.0627	0.321034	-64.0736	0.88653
1	-58.4913	6.7084	-45.2405	1.975258	-67.0046	1.269052	-48.2188	1.531292	-55.1333	0.500566	-61.9533	0.516068

**peak_voltage**												
**Step (nA)**	**Experiment mean**	**Experiment std**	**C10 value**	**C10 error**	**CP15 value**	**CP15 error**	**Tu19 value**	**Tu19 error**	**To21 value**	**To21 error**	**M18 value**	**M18 error**
0.8	23.3497	10.9233	14.4747	0.812483	11.63042	1.072869	12.56237	0.987553	22.16316	0.108625	37.07802	1.256792
1	19.8138	11.8553	9.347181	0.882864	10.53747	0.782463	12.10599	0.650158	19.73156	0.006937	36.45549	1.403734

**spike_half_width**												
**Step (nA)**	**Experiment mean**	**Experiment std**	**C10 value**	**C10 error**	**CP15 value**	**CP15 error**	**Tu19 value**	**Tu19 error**	**To21 value**	**To21 error**	**M18 value**	**M18 error**
0.8	0.7784	0.117	1.906132	9.638731	1.686305	7.759868	1.593291	6.964883	0.715492	0.537673	1.306373	4.512586
1	0.8323	0.1475	1.735355	6.122407	1.59268	5.155117	1.743344	6.176572	0.677314	1.050752	1.314855	3.271556

**spike_width2**

Step (nA)	Experiment mean	Experiment std	C10 value	C10 error	CP15 value	CP15 error	Tu19 value	Tu19 error	To21 value	To21 error	M18 value	M18 error
0.8	0.7538	0.103	1.495442	7.200404			1.467964	6.933634	0.612595	1.370919	1.075016	3.118601
1	0.7911	0.119	1.638763	7.123216	1.40962	5.19765	1.630358	7.052588	0.598542	1.618138	1.085496	2.473916

time_to_first_spike												
**Step (nA)**	**Experiment mean**	**Experiment std**	**C10 value**	**C10 error**	**CP15 value**	**CP15 error**	**Tu19 value**	**Tu19 error**	**To21 value**	**To21 error**	**M18 value**	**M18 error**
0.8	14.1697	13.3451	1.4	0.956883	2.7	0.859469	1.9	0.919416	5	0.687121	4	0.762055
1	8.6998	6.7175	1.2	1.116457	2.2	0.967592	1.6	1.056911	3.6	0.759181	3.1	0.833614

time_to_second_spike												
**Step (nA)**	**Experiment mean**	**Experiment std**	**C10 value**	**C10 error**	**CP15 value**	**CP15 error**	**Tu19 value**	**Tu19 error**	**To21 value**	**To21 error**	**M18 value**	**M18 error**
0.8	29.2121	22.1682	23.1	0.275715			8.5	0.934316	26	0.144897	17.8	0.514796
1	18.8298	10.3516	15.7	0.302349	130.7	10.80704	7	1.142799	15	0.369972	14.1	0.456915

time_to_last_spike												
**Step (nA)**	**Experiment mean**	**Experiment std**	**C10 value**	**C10 error**	**CP15 value**	**CP15 error**	**Tu19 value**	**Tu19 error**	**To21 value**	**To21 error**	**M18 value**	**M18 error**
0.8	302.7331	85.1578	395.5	1.089353	2.7	3.52326	398.3	1.122233	301.8	0.010957	365	0.731194
1	309.4068	100.0108	398.8	0.893835	310.1	0.006931	394.5	0.85084	374.9	0.654861	387.2	0.777848
